# Supplementary material for: Multicentre survey of retinopathy of prematurity in Indonesia
Source: BMJ Paediatr Open. 2021 Jan 22;5(1):e000761. doi: 10.1136/bmjpo-2020-000761 (PMC7831712; doi:10.1136/bmjpo-2020-000761)
Supplement: Supplementary data [file bmjpo-2020-000761supp001.pdf]

Supplementary Table. Incidence of ROP in Indonesia based on the birth weight in 2005-2017

Variable		Inborn infants BW <1000 gram						Inborn infants BW 1000-1500 gram						Inborn infants BW >1500-2000 gram					
		2005-2015	2016-2017					2005-2015	2016-2017					2005-2015	2016-2017				
		HKWCH	RSCM	HKWCH	UBH	OH	All Hospital	HKWCH	RSCM	HKWCH	UBH	OH	All Hospital	HKWCH	RSCM	HKWCH	UBH	OH	All Hospital
Total (Inborn)	n	182	132	71	1024	361	1588	437	306	160	2104	1094	3664	748	475	236	3730	2055	6496
Died	n	85	101	38	567	256	962	72	93	24	640	346	1103	.	42	24	532	205	803
	%	47	77	54	55	71	61	16	30	15	30	32	30	.	9	10	14	10	12
Survived	n	97	31	33	457	105	626	365	213	136	1464	748	2561	.	433	212	3198	1850	5693
Screened	n	59	26	29	197	35	287	252	169	123	802	273	1413	163	177	54	1131	495	1857
Screened/Survived	%	61	84	88	43	33	46	69	79	90	55	36	55	.	41	25	35	27	33
No ROP	n	27	24	26	156	28	234	172	167	120	696	265	1248	139	175	53	488	492	1208
ROP 1-2	n	22	2	2	33	5	42	71	1	3	89	7	100	22	1	1	54	2	58
ROP 3-5	n	10	0	1	6	2	9	9	1	0	17	1	19	2	1	0	0	1	2
Prevalence of Any ROP	%	54	8	10	20	20	18	32	1	2	13	3	8	15	1	2	5	1	3
Prevalence of Severe ROP	%	17	0	3	3	6	3	4	1	0	2	0	1	1	1	0	0	0	0

Note: RSCM/NRH: Ciptomangunkusumo Hospital (National Referral Hospital); HKWCH: Harapan Kita Women and Children Hospital (National Centre for Women and Children's Health); UBH: University Based Hospital; OH: Other Hospital; All: All Hospital; ROP; Retinopathy of Prematurity; BW: Birth weight.
