# Supplementary material for: Multicentre survey of retinopathy of prematurity in Indonesia
Source: BMJ Paediatr Open. 2021 Jan 22;5(1):e000761. doi: 10.1136/bmjpo-2020-000761 (PMC7831712; doi:10.1136/bmjpo-2020-000761)
Supplement: Author's manuscript [file bmjpo-2020-000761.draft_revisions.pdf]

BMJ Paediatrics Open

BMJ Paediatrics Open is committed to open peer review. As part of this commitment we make the peer review history of every article we publish publicly available.

When an article is published we post the peer reviewers' comments and the authors' responses online. We also post the versions of the paper that were used during peer review. These are the versions that the peer review comments apply to.

The versions of the paper that follow are the versions that were submitted during the peer review process. They are not the versions of record or the final published versions. They should not be cited or distributed as the published version of this manuscript.

BMJ Paediatrics Open is an open access journal and the full, final, typeset and author-corrected version of record of the manuscript is available on our site with no access controls, subscription charges or pay-per-view fees (http://bmjpaedsopen.bmj.com).

If you have any questions on BMJ Paediatrics Open's open peer review process please email info.bmjpo@bmj.com

BMJ Paediatrics Open

Indonesian Multicenter Study of ROP, a survey in 34 hospitals

Journal:	*BMJ Paediatrics Open*
Manuscript ID	bmjpo-2020-000761
Article Type:	Original research
Date Submitted by the Author:	18-Jun-2020
Complete List of Authors:	Siswanto, J; Rumah Sakit Anak dan Bunda Harapan Kita, Dep. Neonatology; Pelita Harapan University Faculty of Medicine, Pediatric<br>Bos, Arend F.; University Medical Center Groningen Intensive Care Medicine, Neonatology<br>Dijk, Peter; Universitair Medisch Centrum Groningen, Neonatology<br>Rohsiswatmo, Rinawati ; University of Indonesia Faculty of Medicine, Pediatric<br>Irawan, Gatot; Dr Kariadi General Hospital Medical Center, Pediatric<br>Sulistijono, Eko; Dr Saiful Anwar General Hospital, Pediatric<br>Sianturi, Pertin; University of Sumatera Utara Faculty of Medicine, Pediatric<br>Wisnumurti, Dewi; University of Riau Faculty of Medicine, Pediatric<br>Wilar, Rocky; Sam Ratulangi University Faculty of Medicine, Pediatric<br>Sauer, Pieter; Universitair Medisch Centrum Groningen, Neonatology
Keywords:	Neonatology, Ophthalmology, Epidemiology, Mortality, Health services research

SCHOLARONE™
Manuscripts

I, the Submitting Author has the right to grant and does grant on behalf of all authors of the Work (as defined in the below author licence), an exclusive licence and/or a non-exclusive licence for contributions from authors who are: i) UK Crown employees; ii) where BMJ has agreed a CC-BY licence shall apply, and/or iii) in accordance with the terms applicable for US Federal Government officers or employees acting as part of their official duties; on a worldwide, perpetual, irrevocable, royalty-free basis to BMJ Publishing Group Ltd ("BMJ") its licensees and where the relevant Journal is co-owned by BMJ to the co-owners of the Journal, to publish the Work in this journal and any other BMJ products and to exploit all rights, as set out in our licence.

The Submitting Author accepts and understands that any supply made under these terms is made by BMJ to the Submitting Author unless you are acting as an employee on behalf of your employer or a postgraduate student of an affiliated institution which is paying any applicable article publishing charge ("APC") for Open Access articles. Where the Submitting Author wishes to make the Work available on an Open Access basis (and intends to pay the relevant APC), the terms of reuse of such Open Access shall be governed by a Creative Commons licence – details of these licences and which Creative Commons licence will apply to this Work are set out in our licence referred to above.

Other than as permitted in any relevant BMJ Author's Self Archiving Policies, I confirm this Work has not been accepted for publication elsewhere, is not being considered for publication elsewhere and does not duplicate material already published. I confirm all authors consent to publication of this Work and authorise the granting of this licence.

Confidential: For Review Only

Title: Indonesian Multicenter Study of ROP, a survey in 34 hospitals

First Author/Corresponding Author: **J. Edy Siswanto**

Corresponding Author's Institution:

Department of Neonatology, Harapan Kita Women and Children Hospital

Postal Address:

Rumah Sakit Anak dan Bunda Harapan Kita

Jl. Letjen S. Parman Kav. 87

Jakarta Barat, DKI Jakarta, ID 11420

Indonesia

E-mail: edyjo15@yahoo.com, edy.jos@gmail.com

Telephone number : +62215668284 and +6287786293779

Fax number : +62215657547

Keywords. Retinopathy of Prematurity, incidence, mortality

Word count: 2239

Running title: Incidence of ROP, mortality, Indonesia

Confidential: For Review Only

Indonesian Multicenter Study of ROP, a survey in 34 hospitals

J. Edy Siswanto[1a, b], Arend F. Bos[2],
Peter Dijk[2], Rinawati Rohsiswatmo[3], Gatot Irawan[4], Eko Sulistijono[5], Pertin Sianturi[6],
Dewi A. Wisnumurti[7], Rocky Wilar[8],
Pieter JJ Sauer[2], for the IMSROP study group[*]

Author information

[1a]Neonatology Working Group, Department of Pediatrics, Harapan Kita Women and Children Hospital, Jakarta, Indonesia

[1b]Faculty of Medicine Pelita Harapan University, Tangerang

[2]Department of Pediatrics, Beatrix Children's Hospital, University Medical Center Groningen, Groningen, The Netherlands

[3]Department of Pediatrics, Faculty of Medicine University of Indonesia, Rumah Sakit Cipto Mangunkusumo, Jakarta

[4]Department of Pediatrics, Faculty of Medicine Diponegoro University, RSUP Dr. Kariadi, Semarang, Central Java

[5]Department of Pediatrics, RSUD Dr. Syaiful Anwar, Faculty of Medicine Brawijaya University Malang, East Jawa

[6]Department of Pediatrics, Faculty of Medicine University Sumatera Utara (USU), RSUP H. Adam Malik, Medan, North Sumatera

[7]Department of Pediatrics, RSUD Arifin Achmad Pekanbaru, Faculty of Medicine Riau University

[8]Department of Pediatrics, RSUP Prof. Dr. RD Kandou Hospital, Faculty of Medicine Sam Ratulangi University, Manado, North Sulawesi

Abstract

Background In previous surveys, a higher incidence of Retinopathy of Prematurity (ROP) was found in Indonesia compared with developed countries. A new protocol on how to prevent, screen, and treat ROP was introduced in 2010 to try to reduce the incidence. Thereafter focus meetings were held. National Health Insurance was introduced in 2014.

Objective To evaluate whether the introduction of the guidelines, attention to the high incidence of ROP and national health insurance might have influenced the incidence of ROP in Indonesia.

Setting Data were collected from 34 hospitals with different levels of care, National Referral Centers, University-based Hospitals, Government, and Private Hospitals.

Confidential: For Review Only

Methods A questionnaire was send asking the number of admissions, mortality rate, incidence, and the stage of ROP, disaggregated by gestational age and birth weight. Data were asked for the years 2016-2017.

Results We identified 12,115 eligible infants with a gestational age of less than 34 weeks. Mortality was 24%, any stage ROP 6.7%. The mortality in infants of less than 28 weeks was 67%, the incidence of all stage ROP 18%, and severe ROP 4%. In the group 28-32 weeks, the mortality was 24%, all stage ROP 7%, and severe ROP 4-5%. Mortality, as well as the incidence of ROP, was highest in the University-based hospitals.

Conclusions The mortality rate in infants born before 32 weeks is higher in Indonesia in the period 2016-2017 compared to developed countries. The incidence of ROP is comparable to rates found in developed countries. The incidence of ROP in Indonesia might be an underestimation due to the high mortality. The incidence of ROP in 2016-2017 is lower compared to surveys done before 2015. The reduction in the incidence of ROP in Indonesia might be due to higher awareness about ROP among practitioners and the introduction of a national health care system.

Introduction

Studies done on the incidence of Retinopathy of Prematurity (ROP) during the period 2005-2015 in Indonesia showed a relatively high incidence of ROP.[1, 2] Moreover, ROP was also seen in infants with gestational ages above 32 weeks. In view of these findings, a group of neonatologists and ophthalmologists came together in 2009 and 2010 to develop a guideline for early ROP detection and management for neonatologists and ophthalmologists in Indonesia. This guideline consists of advice on how to prevent ROP and how and when to

Confidential: For Review Only

screen newborn infants. The guideline recommends screening all infants born before 34 weeks or with a birthweight less than 1500 grams, and infants of higher gestational age and higher birth weight who received oxygen for a prolonged time. The national guideline was published in 2010.[3] The introduction of the guideline did not result in national implementation. This is most likely due to a lack of pediatricians' and ophthalmologists' knowledge about ROP and financial constraints in hospitals. Therefore, after the 2010 era, several meetings were organized by the Pediatric and Ophthalmologist Societies in Indonesia to make neonatologists and ophthalmologists aware of the high incidence of ROP, of ways to prevent ROP, and how to perform the screening procedures. Secondly, in 2014 a national health insurance was introduced in Indonesia which increased the possibility of treating premature infants and improving screening for ROP. The care for preterm infants is given in Indonesia in two national referral centers, university-based NICU's as well as Governmental and Private Hospitals. There is a marked variation between the options to care for sick preterm infants between these hospitals. It is not known if these differences between hospitals result in a different incidence of ROP.

In order to evaluate whether the attention asked for the high incidence of ROP in Indonesia and the national health insurance might have reduced the incidence of ROP in Indonesia we conducted a survey on the incidence of ROP in neonatal intensive care centers and local hospitals in Indonesia in the years 2016-2017. As the incidence might be different between hospitals, we collected data from all levels of hospitals where care for sick newborn infants is given.

Methods.

Confidential: For Review Only

A questionnaire was sent to the national referral centers for perinatology, university-based neonatal intensive care centers as well as government and private hospitals. In total 34 hospitals were invited to participate in this survey. In the questionnaire, we asked for the following data: number of inborn preterm infants, number of preterm infants that died in the perinatal period, number of infants screened for ROP, number of infants with ROP, and the stage of ROP. For all data, we asked to specify the data according to gestational age and birth weight. For the analysis, we combined the university-based hospitals and the district (government)/private hospitals in two separate groups. The data for the two national referral perinatal centers are shown separately.

Patient involvement

Patients or the public were not involved in the design, or conduct, or reporting, or dissemination plans of our research

Results.

We received data from the two national referral hospitals for perinatology (Harapan Kita Women and Children Health Center and RSCM- Ciptomangunkusumo Hospital), 14 University Based Hospital NICU'S, and 18 other hospitals, which included 11 government hospitals and 7 private hospitals (table 1).

We received in total data on 12.115 infants with a gestational age <34 weeks. 5252 Infants had a birth weight of less than 1500 g. The overall mortality of infants <34 weeks was 24.1%. Almost 37% of surviving infants were screened for ROP, the incidence of all stage ROP was 6.7%. The highest incidence of both mortality and ROP was found in the University-Based Hospitals (table 1). In table 2 the data are shown for each -group of- hospitals according to

Confidential: For Review Only

gestational age. The overall mortality in the group <28 weeks was 67% without differences between -groups of- hospitals. Sixty-three percent of surviving infants were screened for ROP, the rate of screening varied from 93% in one referral hospital to 42% in the group of other hospitals. The overall rate of any stage ROP was 18%, severe ROP 4%. The incidence of both any stage ROP and severe ROP was higher in the university-based NICU'S and other hospitals compared to both national centers (18-21% vs 4-8%) (figure 1). Severe ROP was not seen in the national centers, while it was seen in 4 and 5% of infants in the university and other hospitals. The same trend was found in infants born after 28-32 weeks. Overall mortality in this group was 24%, with the highest mortality in the university hospitals (31%) compared with 11% in one of the national centers. The rate of screening in the group 28-32 weeks was lower compared to the group <28 weeks, 41 vs 63% and varied between 86% in one national center to 19% in the group other hospitals. Any stage ROP was found in 7% of surviving infants, with the highest incidence (11%) in the university hospitals and 1-2% in the other hospitals. The incidence of severe ROP was 0-1% in all hospitals. The mortality in the group 32-34 weeks was on average 14% with no important differences between hospitals. The rate of screening in these infants was 32%, the prevalence of any stage ROP in this group was 4%, with the highest incidence in university-based hospitals.

In the supplementary table, the data are shown for each -group of- hospitals according to birth weight. The overall mortality in the group <1000 gram was 61% without differences between -groups of- hospitals. Forty-six percent of surviving infants were screened for ROP, the rate of screening varied from 88% in one referral hospital to 33% in the group of other hospitals. The overall rate of any stage ROP was 18%, severe ROP 3%. The incidence of both any stage ROP and severe ROP was higher in the university-based NICU'S and other hospitals compared to both national centers (20% vs 8-10%). Severe ROP was not seen in the national

Confidential: For Review Only

centers, while it was seen in 3 and 6% of infants in the university and other hospitals. The same trend was found in infants born after 1000-1500 grams. Overall mortality in this group was 30%, with the highest mortality in the university and other hospitals (30%) compared with 15% in one of the national centers. The rate of screening in the group 1000-1500 gram was higher compared to the group <1000 gram, 55 vs 46%, and varied between 90% in one national center to 36% in the group other hospitals. Any stage ROP was found in 8% of surviving infants, with the highest incidence (13%) in the university hospitals and 3% in the other hospitals. The incidence of severe ROP was 1% in all hospitals. The mortality in the group >1500-2000 gram was on average 12% with no important differences between hospitals. The rate of screening in these infants was 33%, the prevalence of any stage ROP in this group was 3%, with the highest incidence in university-based hospitals (5%).

In order to evaluate if the rate of screening might have affected the incidence of ROP found in our survey, we calculated the incidence of ROP in 13 out of the 34 hospitals where at least 80% of infants were screened once or more (table 3). There was no difference in the rate of ROP between these hospitals and the total group. In the <28 weeks group, the ROP rate for all stages was 21% and 5% for severe ROP. In the 28-32 weeks group, the incidence of all ROP stages was 6%, severe ROP 1%.

Discussion.

In this survey conducted in 2016-2017, we found that the incidence of any stage ROP in infants born <28 weeks in Indonesia was 18% and 7% in the 28-32 weeks group. The incidence of severe ROP was 4% in the <28 weeks group and 1% in the 28-32 weeks group. The incidence of ROP in Indonesia in the period 2016-2017 is much lower compared to the incidence we found in 2005-2015. We found in this period in one NICU an incidence of all stage ROP of 40%

For Review Only

in infants below 28 weeks and 28% in the group 28-32 weeks.[1] Other studies from this period in Indonesia found in infants born before 32 weeks and/or with a birth weight of less than 1500 gram a ROP incidence of 18-30%.[2] Different factors might have contributed to this marked decrease in ROP. First, a national guideline for the prevention and screening of ROP in Indonesia was published in 2009. When we realized that only publishing this guideline did not change the practice in Indonesia, several focus meetings were held all over Indonesia to ask the attention of practitioners for the high incidence of ROP in Indonesia, the methods to prevent ROP, and the importance of screening.[3] Secondly, in 2014 a national health insurance plan was introduced in Indonesia, improving the possibilities for treating and screening preterm infants. We cannot differentiate which of these factors might have had the most impact on the decrease in the incidence of ROP.

It is difficult to compare the incidence of ROP in Indonesia to the incidence reported in developed countries. A recent study from Greece showed an incidence of any stage ROP in infants <32 weeks of 19.7%, severe ROP 7.4%.[4] In the EXPRESS study from Sweden, the incidence of any stage ROP in infants <31 weeks of 31.9% was found, severe ROP in 5.7%.[5] There is a marked difference, however, between Indonesia and developed countries in the type of infants cared for and the survival of these infants. In Indonesia, almost no infants born after a gestational age of 26 weeks or less, will survive. The mortality in infants of 26-28 weeks in Indonesia is much higher compared to developed countries. In our survey, we found that 67% of infants born <28 weeks, died. Recent data from Sweden, England, France, the Netherlands, Canada, and the USA show the survival of infants born between 26-28 weeks of 80-90 %.[6-16] In Indonesia, only the healthier very preterm infants will survive. In these infants, the incidence of ROP might be lower than in the very sick newborns, who died. The same might be true for infants born after 28-32 weeks. We found that, in Indonesia, 24% of these

Confidential: For Review Only

infants died. In developed countries, this percentage is less than 5%. It might well be that the present data on the incidence of ROP in Indonesia is an underestimation caused by the higher death rate in Indonesia.

Another reason for an underestimation of the true incidence might be the rather low rate of screening. We found however no difference in the incidence of ROP in the institutions with a rate of screening of at least 80%, compared to the whole group of infants. The follow-up of preterm infants admitted to a NICU is in Indonesia, due to socio-economic factors, low. It is not known therefore if ROP was present in surviving, not screened infants. The low rate of screening is due to a lack of ophthalmologists trained in ROP screening.

The incidence of ROP in Harapan Kita Women and Children Hospital was significantly lower in the period 2016-2017 compared to the period 2005-2015. At the same time, the rate of screening improved to 93%. This indicates that ROP decreased significantly in this hospital. In 2016 a strict policy was introduced to set the oxygen saturation monitor for preterm infants between 91% and 95%. The use of oxygen in the delivery room was also strictly regulated, resuscitation of preterm infants was done with in principle 30% oxygen. CPAP was given directly after birth and continued in the NICU. These measures have, most likely, been important in reducing the incidence of ROP in this NICU.

The highest rate of both mortality and ROP was found in the University-Based Hospitals. University-Based Hospitals are referral hospitals, so they might treat the sickest infants, sicker infants than cared for in governmental and private hospitals. This could explain the higher mortality and higher rate of ROP. At the same time, however, the two national centers are also referral hospitals and they showed a lower mortality rate and a lower incidence of ROP (figure 1,2). So, there might be other factors causing the higher incidence of ROP in the

Confidential: For Review Only

University-Based Hospitals. More liberal use of oxygen might be a cause. More studies are needed to determine the cause of the less favorable outcome in the University-Based Hospitals.

A limitation of this study is that the survey on the incidence of ROP as done in this study was not done before. In this paper, we used data from one referral hospital as well as data published in the literature as a reference. The rates of ROP found in one hospital in Indonesia[1] as well as the studies done in other centers[2], all showed a high incidence of ROP in the period before 2015. Therefore, we are convinced that the incidence of ROP was indeed much higher compared to the results found in this survey.

Conclusion.

The incidence of ROP in preterm infants in Indonesia was lower in 2016-2017 compared to the period before 2015. This might be due to a higher awareness of ROP among practitioners and to the introduction of a national health care system. Our data, however, might be an underestimation of the real incidence of ROP due to the higher mortality rate in small preterm infants in Indonesia compared to developed countries.

Acknowledgements The authors would like to thank all infants and families whose data contribute to the IMSROP Study Group. We thank Dr. Florence Manurung, Dr. Habsyiyah, and Dr. Nani H. Widodo, as well as all the ophthalmology colleagues involved in each hospital included in this study, were involved in examining patients and providing scientific input in the field of ophthalmology and Prof. Rita S. Sitorus who encouraged us to move hand in hand

Confidential: For Review Only

together to reduce the incidence of ROP in Indonesia in terms of neonatology, ophthalmology, and public health.

Collaborators

*Members of the IMSROP (Indonesian Multicenter Retinopathy of Prematurity) Study Group: Rinawati Rohsiswatmo *(Rumah Sakit Cipto Mangunkusumo, Jakarta Pusat)*, Tetty Yuniati *(RSUP Hasan Sadikin, Bandung, Jawa Barat)*, Gatot Irawan Sarosa *(RSUP dr. Kariadi, Semarang, Jawa Tengah)*, Dwi Hidayah *(RSUD Dr. Moewardi, Solo, Jawa Tengah),* Dina Angelika dan Risa Etika *(RSUD Dr. Soetomo Surabaya, Jawa Timur)*, Eko Sulistijono *(RSUD Dr. Syaiful Anwar Malang, Jawa Timur)*, Made Kardana *(RSUP Sanglah, Denpasar, Bali)*, Pertin Sianturi *(RSUP H. Adam Malik, Medan, Sumatera Utara)*, Eni Yantri *(RSUP Dr. M. Djamil Padang, Sumatera Barat)*, Dewi Anggraini Wisnumurti *(RSUD Arifin Achmad Pekanbaru, Riau)*, Afifa Ramadanti *(RSUP M. Hoesin Palembang, Sumatera Selatan)*, Pudji Andayani *(RSUD Ulin Banjarmasin Kalimantan Selatan)*, Rocky Wilar *(RSUP Prof. dr. RD Kandou Hospital, Manado)*, Ema Alasiry (RSUP Dr. Wahidin Sudirohusodo, Makasar, Sulawesi Selatan), Harris Alfan *(RS Atma Jaya, Jakarta Utara)*, Johanes Edy Siswanto *(RSAB Harapan kita dan RS Royal Taruma, Jakarta Barat)*, Naomi Esthernita *(Siloam Hospital Kebon Jeruk, Jakarta Barat)*, Fahrul Wakil Arbi *(RS Budi Kemuliaan, Jakarta Pusat)*, Nita Dewanti *(RS Premier Bintaro, Jakarta Selatan)*, Agnes Yuni Purwita Sari *(RSUP Persahabatan, Jakarta Timur)*, Ellen Sianipar *(RSUD Pasar Rebo Jakarta Timur)*, Robert Soetandio *(RS Awal Bros Hospital, Tangerang, Jawa Barat)*, Andika Tiurmaida *(RSUD Cibinong, Jawa Barat)*, Thomas Harry Adoe *(RSUD Kota Bekasi)*, Muda Isa Ariantana *(Santosa Hospital Bandung Cermai Bandung, Jawa Barat)*, Samad *(RSUP Dr. Soeradji Tirtonegoro Klaten),* Anince *(RS Bakti Timah Karimun, Kepulauan Riau)*, Tri Putri Yuliani *(RSUD Siti Aisyah Kota Lubuk Linggau)*, Sumsel, Dina Frida *(RSUD Dr. Sudarso, Kalimantan Barat dan RS Anugrah Bunda Khatulistiwa, Kalimantan Barat)*, Nurhandini Eka Dewi *(RSUD Mataram, Nusa Tenggara Barat)*, Woro Indri P. Purba *(RSUD Prof. Dr. WZ Johannes Kupang, Nusa Tenggara Timur),* Sadiyah *(RSUD I Lagaligo, Luwu Timur, Sulawesi Selatan)*.

Contributors JES is the person who involved directly in whole aspects of this research and manuscript. JES, AB, PD, and PJJS contributed to study design and analysis. PJJS assisted in the literature review, wrote the report, and wrote the discussion section. AFB and PJJS are the guarantors for the study. JES, AB, and PJJS had full access to the IMSROP data. All authors had full access to the summary data presented in this paper (including statistical reports and

tables) in the study and can take responsibility for the integrity of the data and the accuracy of the data analysis.

Funding The authors have not declared a specific grant for this research from any funding agency in the public, commercial, or not-for-profit sectors.

Competing interests None declared.

Patient consent Detail has been removed from this case description/these case descriptions to ensure anonymity. The editors and reviewers have seen the detailed information available and are satisfied that the information backs up the case the authors are making.

Ethics approval The study was approved by the Institutional Review Board of the University of Indonesia School of Public Health (No. 32/H2.F10/PPM.00/13)

Provenance and peer review Not commissioned; externally peer-reviewed.

What is already known on this topic?

► Several studies in the previous decade in Indonesia showed a high incidence of ROP which is a serious problem as in other LMIC countries.

► The high rate of ROP in Indonesia is most likely due to an expansion of neonatal intensive care while the awareness of the risks to develop ROP is lacking.

What this study adds?

►The incidence of ROP in Indonesia was lower in 2016-2017 compared to surveys conducted before 2015.

Confidential: For Review Only

► There is a difference in mortality and rate of ROP between the types of hospitals. Mortality, as well as the incidence of ROP, was highest in University-based hospitals.

► The lower rate of ROP might be the result of a new national guideline on ROP, of an increased awareness due to focus meetings and the introduction of national health care.

Confidential: For Review Only

References

1. Siswanto JE, Nani H Widodo NH, Sauer PJJ. *Arch Dis Child 2018*;103:619–21.
2. Siswanto JE, Sauer PJ. Retinopathy of prematurity in Indonesia: Incidence and riskfactors. J *Neonatal Perinatal Med* 2017;10:85–90.
3. Sitorus RS et al. POKJA Nasional ROP dan Bayi Prematur, Pedoman Nasional: Skrining dan Terapi ROP pada bayi prematur di Indonesia. eds 1. 2010
4. Mataftsi A, Moutzouri S, Karagianni P, et al. Retinopathy of prematurity occurrence and evaluation of screening policy in a large tertiary Greek cohort. *Int Ophthalmol* 2020;40:385-91
5. Austeng D, Källen K, Hellström A, et al. Regional differences in screening for retinopathy of prematurity in infants born before 27 weeks of gestation in Sweden--the EXPRESS study. *Acta Ophthalmol* 2014; 92:311-5.
6. Draper ES, Zeitlin J, Field DJ, et al. Mortality patterns among very preterm babies: A comparative analysis of two European regions in France and England. *Arch Dis Child Fetal Neonatal Ed* 2007; 92: 356–360.
7. Milligan DW. Outcomes of children born very preterm in Europe. *Arch Dis Child Fetal Neonatal Ed* 2010;95:F234-40.
8. Draper ES, Zeitlin J, Fenton AC, et al, on behalf of the MOSAIC research group. Investigating the variations in survival rates for very preterm infants in 10 European regions: the MOSAIC birth cohort. *Arch Dis Child Fetal Neonatal Ed* 2009;3:F158-63.
9. Mohangoo AD, Buitendijk SE, Szamotulska K, et al. Gestational Age Patterns of Fetal and Neonatal Mortality in Europe: Results from the Euro-Peristat Project. *PLoS ONE* 2011;6:e24727.
10. Serenius F, Sjörs G, Blennow M, et al, and For the EXPRESS study group*. EXPRESS study shows significant regional differences in 1-year outcome of extremely preterm infants in Sweden. *Acta Paediatr* 2014;103:27–37.
11. Santhakumaran S, Statnikov Y, Gray D, et al, on behalf of the Medicines for Neonates Investigator Group. Survival of very preterm infants admitted to neonatal care in England 2008–2014: time trends and regional variation. *Arch Dis Child Fetal Neonatal Ed* 2018;103:F208–F215.

12. Norman M, Hallberg B, Abrahamsson T, et al. Association Between Year of Birth and 1-Year Survival Among Extremely Preterm Infants in Sweden During 2004-2007 and 2014-2016. *JAMA* 2019; 321:1188-1199.
13. Lee SK, McMillan DD, Ohlsson A, et al. Variations in practice and outcomes in the Canadian NICU network: 1996-1997. *Pediatrics* 2000;106:1070–9.
14. Stoll BJ, Hansen NI, Bell EF, et al. Neonatal Outcomes of Extremely Preterm Infants From the NICHD Neonatal Research Network. *Pediatrics* 2010; 126: 443–456.
15. Stoll BJ, Hansen NI, Bell EF, et al. Trends in Care Practices, Morbidity, and Mortality of Extremely Preterm Neonates, 1993-2012. *JAMA* 2015;314:1039-51.
16. Su BH, Hsieh WS, Hsu CH, Chang JH, Lien R, Lin CH; Premature Baby Foundation of Taiwan (PBFT). Neonatal Outcomes of Extremely Preterm Infants From Taiwan: Comparison With Canada, Japan, and the USA. *Pediatr Neonatol* 2015;56:46-52.

Figure legend

Figure 1. The prevalence of ROP based on gestational age in each group of the hospital in Indonesia 2016-2017.

Figure 2. Mortality based on the gestational age in each group of the hospital in Indonesia 2016-2017

Confidential: For Review Only

Table 1. Hospital-based ROP data surveillance in Indonesia 2016-2017 (GA ≤ 34 weeks)

Type of hospital	Total Hospital	Total Infants	Survived	Died		Screened		ROP	
				n	%	n	%	n	%
RSCM/NRH	1	1038	890	148	14,26	392	44,04	7	1,79
HKWCHC	1	478	392	86	17,99	281	71,68	7	2,49
UBH	14	6549	4738	1811	27,65	2041	43,08	197	9,65
OH	18	4050	3261	789	19,48	711	21,80	17	2,39
- Government Hospital	10	2155	1560	595	27,61	605	38,78	15	2,48
- Private Hospital	8	1030	836	194	18,83	106	12,68	2	1,89
All Hospital	34	12115	9281	2834	23,39	3425	36,90	228	6,66

Note:
RSCM/NRH: Ciptomangunkusumo Hospital (National Referral Hospital); HKWCH: Harapan Kita Women and Children Hospital (Health Centre); UBH: University Based Hospital; OH: Other Hospital; All: All Hospital; ROP; Retinopathy of Prematurity.

Confidential: For Review Only

Table 2. ROP prevalence in Indonesia based on the gestational age in 2005-2017

Variable		Inborn infants GA <28 weeks						Inborn infants GA 28 - 32 weeks						Inborn infants GA >32 - 34 weeks					
		2005-2015	2016-2017					2005-2015	2016-2017					2005-2015	2016-2017				
		HKWCH	RSCM	HKWCH	UBH	OH	All	HKWCH	RSCM	HKWCH	UBH	OH	All	HKWCH	RSCM	HKWCH	UBH	OH	All
Total (Inborn)	n	185	115	54	903	354	1426	569	501	221	2288	2044	5054	NA	422	203	3358	1652	5635
Died	n	91	87	27	577	259	961	126	122	24	700	349	1195	-	26	24	534	181	765
	%	49	76	50	64	73	67	22	24	11	31	17	24	-	6	12	16	11	14
Survived	n	94	28	27	326	95	465	443	379	197	1588	1695	3859	-	396	179	2824	1471	4870
Screened	n	47	23	25	204	40	292	261	187	169	923	315	1594	156	182	87	914	356	1539
Screened/Survived	%	50	82	93	63	42	63	59	49	86	58	19	41	-	46	49	32	24	32
No ROP	n	28	22	23	161	33	239	187	185	167	751	309	1478	126	178	84	441	352	1480
ROP 1-2	n	9	1	2	34	5	42	66	2	1	94	6	103	29	2	3	45	2	52
ROP 3-5	n	10	0	0	9	2	11	8	0	1	12	0	13	1	2	0	3	2	7
Prevalence Any ROP	%	40	4	8	21	18	18	28	1	1	11	2	7	19	2	3	5	1	3.8
Prevalence Severe ROP	%	21	0	0	4	5	4	3	0	1	1	0	1	1	1	0	0	1	0.4

Note: RSCM/NRH: Ciptomangunkusumo Hospital (National Referral Hospital); HKWCH: Harapan Kita Women and Children Hospital (Health Centre); UBH: University Based Hospital; OH: Other Hospital; All: All Hospital; ROP; Retinopathy of Prematurity; GA: Gestational Age.

Confidential: For Review Only

Table 3. Incidence of ROP in hospitals with 80% screening coverage in Indonesia, 2016-2017

Variable		Birth weight					Gestational age				
		<1000 g	1000-1500 g	>1500-2000 g	>2000-<2500 g	≥2500 g	<28 wks	28-32 wks	32-34 wks	34-<37 wks	≥37 wks
**Total (Inborn)**	n	585	1541	2679	4522	20572	560	2144	2319	3913	20963
**Died**	n	394	449	303	163	266	391	508	244	165	267
	%	67	29	11	4	1	70	24	11	4	1
**Survived**	n	191	1092	2376	4359	20306	169	1636	2075	3748	20696
**Screened**	n	172	1040	1033	1186	14	168	1147	926	1199	5
**Screened/Survived**	%	90	95	43	27	0	99	70	45	32	0
**No ROP**	n	137	966	978	1159	14	132	1074	874	1169	5
**ROP 1-2**	n	28	57	53	27	0	27	62	46	30	0
**ROP 3-5**	n	7	17	2	0	0	9	11	6	0	0
**Prevalence of Any ROP**	%	20	7	5	2	0	21	6	6	3	0
**Prevalence of Severe ROP**	%	4	2	0	0	0	5	1	1	0	0

Note : ROP: Retinopathy of Prematurity

Confidential: For Review Only

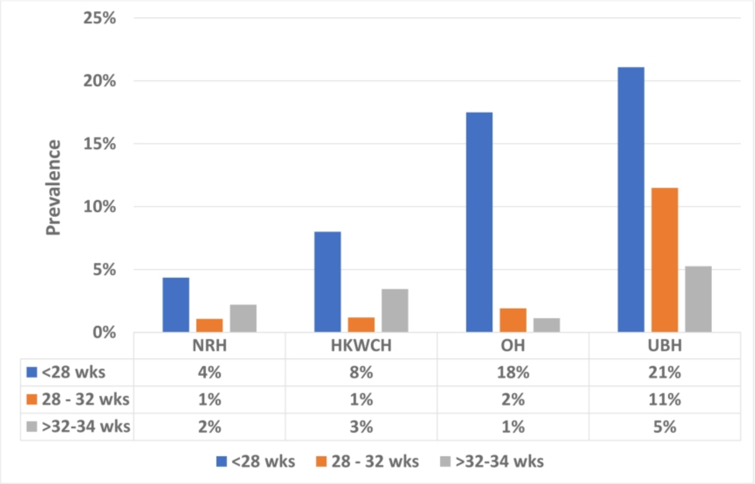

Note: RSCM/NRH: Ciptomangunkusumo Hospital (National Referral Hospital); HKWCH: Harapan Kita Women and Children Hospital (Health Centre); UBH: University Based Hospital; OH: Other Hospital.

148x105mm (1200 x 1200 DPI)

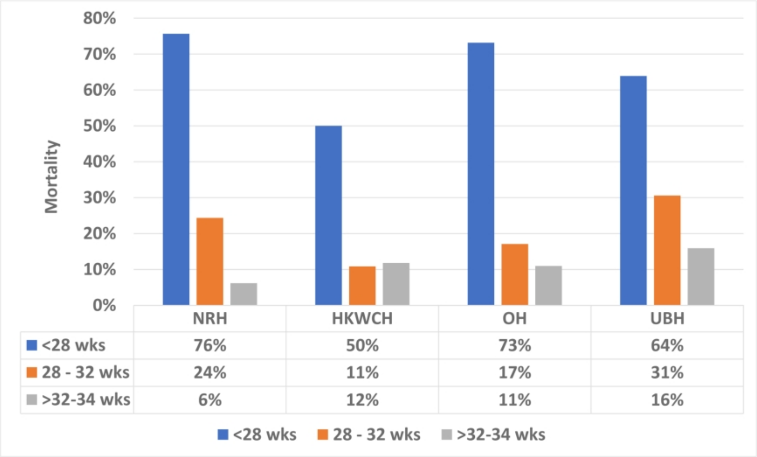

Note: RSCM/NRH: Ciptomangunkusumo Hospital (National Referral Hospital); HKWCH: Harapan Kita Women and Children Hospital (Health Centre); UBH: University Based Hospital; OH: Other Hospital.

148x105mm (1200 x 1200 DPI)

Supplementary Table. Prevalence of ROP in Indonesia based on the birth weight in 2005-2017

Variable		Inborn infants BW <1000 gram						Inborn infants BW 1000 - 1500 gram						Inborn infants BW >1500 - 2000 gram					
		2005-2015	2016-2017					2005-2015	2016-2017					2005-2015	2016-2017				
		HKWCH	RSCM	HKWCH	UBH	OH	All	HKWCH	RSCM	HKWCH	UBH	OH	All	HKWCH	RSCM	HKWCH	UBH	OH	All
Total (Inborn)	n	182	132	71	1024	361	1588	437	306	160	2104	1094	3664	748	475	236	3730	2055	6496
Died	n	85	101	38	567	256	962	72	93	24	640	346	1103	.	42	24	532	205	803
	%	47	77	54	55	71	61	16	30	15	30	32	30	.	9	10	14	10	12
Survived	n	97	31	33	457	105	626	365	213	136	1464	748	2561	.	433	212	3198	1850	5693
Screened	n	59	26	29	197	35	287	252	169	123	802	273	1413	163	177	54	1131	495	1857
Screened/Survived	%	61	84	88	43	33	46	69	79	90	55	36	55	.	41	25	35	27	33
No ROP	n	27	24	26	156	28	234	172	167	120	696	265	1248	139	175	53	488	492	1208
ROP 1-2	n	22	2	2	33	5	42	71	1	3	89	7	100	22	1	1	54	2	58
ROP 3-5	n	10	0	1	6	2	9	9	1	0	17	1	19	2	1	0	0	1	2
Prevalence Any ROP	%	54	8	10	20	20	18	32	1	2	13	3	8	15	1	2	5	1	3
Prevalence Severe ROP	%	17	0	3	3	6	3	4	1	0	2	0	1	1	1	0	0	0	0

Note:
RSCM/NRH: Ciptomangunkusumo Hospital (National Referral Hospital); HKWCH: Harapan Kita Women and Children Hospital (Health Centre);
UBH: University Based Hospital; OH: Other Hospital; All: All Hospital; ROP: Retinopathy of Prematurity; BW: Birth weight.

Confidential: For Review Only

BMJ Paediatrics Open

A multicentre survey of retinopathy of prematurity in Indonesia

Journal:	*BMJ Paediatrics Open*
Manuscript ID	bmjpo-2020-000761.R1
Article Type:	Original research
Date Submitted by the Author:	17-Aug-2020
Complete List of Authors:	Siswanto, J; Rumah Sakit Anak dan Bunda Harapan Kita, Dep. Neonatology; Pelita Harapan University Faculty of Medicine, Pediatric<br>Bos, Arend F.; University Medical Center Groningen Intensive Care Medicine, Neonatology<br>Dijk, Peter; Universitair Medisch Centrum Groningen, Neonatology<br>Rohsiswatmo, Rinawati ; University of Indonesia Faculty of Medicine, Pediatric<br>Irawan, Gatot; Dr Kariadi General Hospital Medical Center, Pediatric<br>Sulistijono, Eko; Dr Saiful Anwar General Hospital, Pediatric<br>Sianturi, Pertin; University of Sumatera Utara Faculty of Medicine, Pediatric<br>Wisnumurti, Dewi; University of Riau Faculty of Medicine, Pediatric<br>Wilar, Rocky; Sam Ratulangi University Faculty of Medicine, Pediatric<br>Sauer, Pieter; Universitair Medisch Centrum Groningen, Neonatology
Keywords:	Neonatology, Ophthalmology, Epidemiology, Mortality, Health services research

SCHOLARONE™
Manuscripts

I, the Submitting Author has the right to grant and does grant on behalf of all authors of the Work (as defined in the below author licence), an exclusive licence and/or a non-exclusive licence for contributions from authors who are: i) UK Crown employees; ii) where BMJ has agreed a CC-BY licence shall apply, and/or iii) in accordance with the terms applicable for US Federal Government officers or employees acting as part of their official duties; on a worldwide, perpetual, irrevocable, royalty-free basis to BMJ Publishing Group Ltd ("BMJ") its licensees and where the relevant Journal is co-owned by BMJ to the co-owners of the Journal, to publish the Work in this journal and any other BMJ products and to exploit all rights, as set out in our licence.

The Submitting Author accepts and understands that any supply made under these terms is made by BMJ to the Submitting Author unless you are acting as an employee on behalf of your employer or a postgraduate student of an affiliated institution which is paying any applicable article publishing charge ("APC") for Open Access articles. Where the Submitting Author wishes to make the Work available on an Open Access basis (and intends to pay the relevant APC), the terms of reuse of such Open Access shall be governed by a Creative Commons licence – details of these licences and which Creative Commons licence will apply to this Work are set out in our licence referred to above.

Other than as permitted in any relevant BMJ Author's Self Archiving Policies, I confirm this Work has not been accepted for publication elsewhere, is not being considered for publication elsewhere and does not duplicate material already published. I confirm all authors consent to publication of this Work and authorise the granting of this licence.

Confidential: For Review Only

Title: A multicentre survey of retinopathy of prematurity in Indonesia

First Author/Corresponding Author: **J. Edy Siswanto**

Corresponding Author's Institution:

Department of Neonatology, Harapan Kita Women and Children's Hospital

Postal Address:

Rumah Sakit Anak dan Bunda Harapan Kita

Jl. Letjen S. Parman Kav. 87

Jakarta Barat, DKI Jakarta, ID 11420

Indonesia

Email: edyjo15@yahoo.com, edy.jos@gmail.com

Telephone number: +62215668284 and +6287786293779

Fax number: +62215657547

Keywords: Retinopathy of Prematurity, incidence, mortality

Word count: ~~2239~~

Running title: Incidence of ROP, mortality, Indonesia

Confidential: For Review Only

A multicentre survey of retinopathy of prematurity in Indonesia

J. Edy Siswanto[1a, b], Arend F. Bos[2],
Peter Dijk[2], Rinawati Rohsiswatmo[3], Gatot Irawan[4], Eko Sulistijono[5], Pertin Sianturi[6],
Dewi A. Wisnumurti[7], Rocky Wilar[8],
Pieter JJ Sauer[2], for the IMSROP study group[*]

Author information

[1a]Neonatology Working Group, Department of Pediatrics, Harapan Kita Women and Children's Hospital, Jakarta, Indonesia

[1b]Faculty of Medicine, Pelita Harapan University, Tangerang

[2]Department of Pediatrics, Beatrix Children's Hospital, University Medical Center Groningen, Groningen, The Netherlands

[3]Department of Pediatrics, Faculty of Medicine, University of Indonesia, Rumah Sakit Cipto Mangunkusumo, Jakarta

[4]Department of Pediatrics, Faculty of Medicine, Diponegoro University, RSUP Dr Kariadi, Semarang, Central Java

[5]Department of Pediatrics, RSUD Dr Syaiful Anwar, Faculty of Medicine, Brawijaya University Malang, East Jawa

[6]Department of Pediatrics, Faculty of Medicine, University Sumatera Utara (USU), RSUP H. Adam Malik, Medan, North Sumatera

[7]Department of Pediatrics, RSUD Arifin Achmad Pekanbaru, Faculty of Medicine, Riau University

[8]Department of Pediatrics, RSUP Prof. Dr RD Kandou Hospital, Faculty of Medicine, Sam Ratulangi University, Manado, North Sulawesi

Abstract

Background The incidence of retinopathy of prematurity (ROP) is higher in Indonesia than in high-income countries. In order to reduce the incidence of the disease, a protocol on preventing, screening and treating ROP was published in Indonesia in 2010. To assist the practical implementation of the protocol, meetings were held in all regions of Indonesia, calling attention to the high incidence of ROP and the methods to reduce it. In addition, national health insurance was introduced in 2014, making ROP screening and treatment accessible to more infants.

Confidential: For Review Only

Objective To evaluate whether the introduction of both the guideline drawing attention to the high incidence of ROP and the national health insurance may have influenced the incidence of the disease in Indonesia.

Setting Data were collected from 34 hospitals with different levels of care: national referral centres, university-based hospitals and public and private hospitals.

Methods A retrospective survey was administered with questions on admission numbers, mortality rates, the incidence of ROP and its stages for 2016-2017 in relation to gestational age and birth weight.

Results We identified 12,115 eligible infants with a gestational age of less than 34 weeks. Mortality was 24% and any-stage ROP 6.7%. The mortality in infants aged less than 28 weeks was 67%, the incidence of all-stage ROP 18% and severe ROP 4%. In the 28-32 week group, the mortality was 24%, all-stage ROP 7% and severe ROP 4-5%. Both mortality and the incidence of ROP were highest in university-based hospitals.

Conclusions In the 2016-2017 period, the infant mortality rate before 32 weeks of age was higher in Indonesia than in high-income countries but the incidence of ROP was comparable. This incidence is likely an underestimation due to the high mortality rate. The ROP incidence in 2016-2017 is lower than in surveys conducted before 2015. This decline is likely due to a higher practitioner awareness about ROP and the implementation of national health insurance in Indonesia.

Introduction

Studies on the incidence of retinopathy of prematurity (ROP) during the period 2005-2015 in Indonesia showed a relatively high incidence of ROP.[1, 2] ROP was also seen in infants with gestational ages above 32 weeks. In view of these findings, a group of neonatologists and

Confidential: For Review Only

ophthalmologists met in 2009 and 2010 to develop a guideline for the prevention, early detection and management of ROP for neonatologists and ophthalmologists in Indonesia. The national guideline, which was published in 2010, provides advice on how to prevent ROP and how and when to screen newborn infants.[3] It recommends screening all infants born before 34 weeks or with a birthweight of less than 1500 g, as well as infants of higher gestational age and higher birth weight who received oxygen for a prolonged period. Neonatologists and paediatric ophthalmologists realized that the guideline's publication had almost no impact on clinical practice. This was most likely due to a lack of knowledge about ROP among paediatricians and ophthalmologists and to financial constraints in hospitals.

After 2010, the paediatric and ophthalmologist societies in Indonesia therefore organized several meetings to alert their members to the high incidence of ROP and to ways to prevent the disease and perform screening procedures. In addition, national health insurance was introduced in 2014, thereby increasing opportunities to treat premature infants and to improve ROP screening. Care for preterm infants in Indonesia is provided by two national referral centres, university-based NICUs (neonatal intensive care centres), as well as governmental and private hospitals. There are marked variations among these hospitals, such as patients' socioeconomic and cultural backgrounds and other demographic factors, in terms of their options for caring for sick preterm infants. We do not know whether the differences between hospitals resulted in a different incidence of ROP.

To evaluate whether an awareness of the high incidence of ROP and the introduction of national health insurance may have reduced that incidence, we conducted a survey on the incidence of the disease at Indonesian neonatal intensive care centres and local hospitals in

Confidential: For Review Only

the years 2016-2017. As the incidence may differ between hospitals, we collected data from all levels of hospitals that provide care for sick newborns.

Methods

This is a retrospective survey: we collected data for the years 2016-2017 in the period from March to November 2019. Paediatricians in 47 hospitals were contacted by email and direct phone calls; 41 were willing to send us the required information. We received responses from 34 hospitals in 17 major provinces of Indonesia – 16 teaching hospitals, two of which are national referral hospitals for perinatology, and 10 government and eight private hospitals. The availability of NICU beds varied greatly across between regions because of a lack of trained neonatologists and differences in stakeholder support in the province or district where the paediatricians worked. We approached hospitals offering all levels of neonatal care, located in all the different parts of Indonesia.

The survey asked for the following data: number of inborn preterm infants, number of preterm infants who died in the perinatal period, number of infants screened for ROP, number of infants with ROP and the stage of ROP. For the sake of uniformity and to analyse all the data, we asked for inborn babies to be further categorised by gestational age and birth weight. For ROP, we used the terms mild ROP (stages 1-2) and severe ROP (stage 3 or higher). We included only inborn infants because important data such as gestational age and complications in pregnancy are often not available for outborn infants. We analysed the university-based hospitals and the district (government)/private hospitals in two separate groups. The data for the two national referral perinatal centres are shown separately. The results are described in frequencies and percentages. No advance statistical test was used for the data analysis.

Confidential: For Review Only

Patient involvement

Neither patients nor the public were involved in the design, execution, reporting or dissemination plans for our research.

Results

We received data from the two national referral hospitals for perinatology (Harapan Kita Women and Children's Health Centre and RSCM-Ciptomangunkusumo Hospital), 14 university-based hospital NICUs and 18 other hospitals, which included 10 government hospitals and eight private hospitals (Table 1).

In total, we received data on 12,115 infants with a gestational age <34 weeks, 5,252 of whom had a birth weight of less than 1500 g. The overall mortality of infants <34 weeks was 24.1%. Almost 37% of surviving infants were screened for ROP; the incidence of all-stage ROP was 6.7%. The highest incidence of both mortality and ROP was found the university-based hospitals (Table 1).

Table 2 shows the data for each hospital or group of hospitals according to gestational age. The overall mortality in the group <28 weeks was 67%, with no differences between hospitals. Sixty-three percent of surviving infants were screened for ROP. The rate of screening ranged from 93% in one referral hospital to 42% in the 'other hospitals' group. The overall rate of any-stage ROP was 18% and severe ROP 4%. The incidence of both any-stage ROP and severe ROP was higher in the university-based NICUs and other hospitals than in the two national centres (18 and 21% vs 4 and 8%) (Figure 1). Severe ROP was not seen in the national centres, but was found in 4% and 5% of infants in the university and other hospitals respectively. The same trend was found in infants born after 28-32 weeks. Overall mortality in this group was

Confidential: For Review Only

24%, with the highest mortality in the university hospitals (31%), compared with 11% in one of the national centres. The rate of screening in the 28-32 week group was lower than in the <28 week group (41 vs 63%) and ranged from 86% in one national centre to 19% in the 'other hospitals' group. Any stage ROP was found in 7% of surviving infants, with the highest incidence (11%) in university hospitals and 1-2% in other hospitals. The incidence of severe ROP was 0-1% in all hospitals. The mortality in the 32-34 week group was 14% on average, with no important differences between hospitals. The rate of screening in these infants was 32% and the prevalence of any stage ROP was 4%, with the highest incidence in university-based hospitals.

The supplementary table shows the data for each hospital or group of hospitals according to birth weight. The overall mortality in the <1000 g group was 61%, with no differences between groups of hospitals. Forty-six percent of surviving infants were screened for ROP; the rate of screening ranged from 88% in one referral hospital to 33% in the 'other hospitals' group. The overall rate of any stage ROP was 18% and severe ROP 3%. The incidence of both any stage ROP and severe ROP was higher in the university-based NICUs and other hospitals than in the two national centres (20% vs 8 and 10%). Severe ROP was not encountered in the national centres but was seen in 3% and 6% respectively of infants in the university and other hospitals. The same trend was found in infants born with a birth weight of 1000-1500 g. Overall mortality in this group was 30%, with the highest mortality in the university and other hospitals (30%), compared with 15% in one of the national centres. The rate of screening in the 1000-1500 g group was higher than in the <1000 g group (55% vs 46%), and ranged from 90% in one national centre to 36% in the 'other hospitals' group. Any-stage ROP was found in 8% of surviving infants, with the highest incidence (13%) in the university hospitals and 3% in other hospitals. The incidence of severe ROP was 1% in all hospitals. The mortality in the

>1500-2000 g group was 12% on average, with no significant differences between hospitals. The rate of screening in these infants was 33% and the prevalence of any stage ROP was 3%, with the highest incidence in university-based hospitals (5%).

In order to assess whether the screening rate may have affected the incidence of ROP found in our survey, we calculated the incidence of ROP in 13 of the 34 hospitals where at least 80% of infants were screened once or more (table 3). There was no difference in the rate of ROP between these hospitals and the total group. In the <28 weeks group, the ROP rate at all stages was 21% and 5% for severe ROP. In the 28-32 weeks group, the incidence of all ROP stages was 6% and severe ROP 1%.

Discussion

Globally, ROP is one of the hidden causes of morbidity following death-related problems such as respiratory distress (asphyxia), infections and other complications that arise in small, sick preterm infants. Countries with a high rate of premature births will consequently see a higher number of infants affected by ROP. This situation occurs in almost all parts of the world, both in high-income (HIC) and lower middle income countries (LMIC).[4]

What has been characterised as the third ROP epidemic has particularly affected countries in Southeast Asia. It is clear that many cases of visual impairment due to ROP are preventable through improved neonatal care, timely retinal examination and appropriate treatment.[5] In this survey, we present data on the incidence and severity of ROP following the implementation of a national guideline for the prevention, screening and treatment of ROP and the introduction of national health insurance in Indonesia, one of the major regions in Southeast Asia.

Confidential: For Review Only

This survey conducted in 2016-2017 found the incidence of ROP in Indonesia in that period to be much lower than the incidence we found in 2005-2015.[1,2] In that previous period, we found in one NICU a 40% incidence of all-stage ROP in infants below 28 weeks and a 28% incidence in the 28-32 week group.[1] Other studies from the period 2005-2015 in Indonesia found a ROP incidence of 18-30% in infants born before 32 weeks and/or with a birth weight of less than 1500 g.[2] Different factors may have contributed to this marked decline in ROP. Firstly, a national guideline for ROP prevention and screening was published in 2010. When we realized that simply publishing this guideline did not change practices in Indonesia, several focus meetings were held across the country to alert practitioners to the high incidence of ROP, the methods to prevent ROP and the importance of screening.[3] In addition, national health insurance was introduced in 2014, thereby improving opportunities for treating and screening preterm infants. We cannot identify which of these factors may have had the greatest impact on the decline in incidence of ROP.

It is difficult to compare the incidence of ROP in Indonesia with the incidence reported in HIC. A recent study from Greece showed an incidence of any stage ROP in infants <32 weeks of 19.7% and severe ROP 7.4%.[6] The EXPRESS study from Sweden found an incidence of any stage ROP in infants <31 weeks of 31.9% and severe ROP 5.7%.[7] There is a marked difference, however, between Indonesia and HIC in the type of infants cared for and their survival. In Indonesia, almost no infants born after a gestational age of 26 weeks or less will survive. Mortality among infants of 26-28 weeks is much higher than in HIC. In our survey, we found that 67% of infants born <28 weeks died. Recent data from Sweden, England, France, the Netherlands, Canada and the USA show a survival rate for infants born between 26-28 weeks of 80-90%.[8-18] In Indonesia, only the healthier very preterm infants will survive. The incidence of ROP may be lower in these infants than in the very sick newborns who died. The same may

Confidential: For Review Only

be true of infants born after 28-32 weeks. In Indonesia, we found that 24% of these infants died. In HIC, that figure is less than 5%. The present data on the incidence of ROP in Indonesia could be an underestimation caused by the higher death rate in Indonesia.

Studies conducted in other LMIC up to 2015 also showed a higher incidence of ROP than in HIC. In addition, ROP was seen in infants with a higher gestational age and birth weight. A study from the Philippines showed a ROP incidence of 14% in all infants born before 36 weeks.[19] A small study from Brunei showed a prevalence of 35% in infants with a birth weight of 1300 ± 500 g and a gestational age of 29.5 ± 2.6 weeks.[20] In Thailand, a ROP incidence of 14% was found in infants with a mean birthweight of 1514 g and a gestational age of 31.8 weeks.[21] In line with our findings for the period 2005-2015, these data indicate that ROP is prevalent in LMIC, including in infants with a higher birthweight and gestational age. A recent paper describes the current state of ROP in eight LMIC.[22] The incidence of ROP was not available for all countries. This incidence, mostly based on smaller studies in one institution, ranged from 14% to 50%. In almost all countries, infants up to 34 weeks and with a birthweight of 2000 g were screened. A study from Thailand, where only infants born <30 weeks and with a birthweight of <1500 g were screened, found a ROP incidence of 40%. In all countries, the screening rate was low, at <35%. The reasons mentioned for the high incidence of ROP was similar for all countries: a lack of awareness among paediatricians, a shortage of trained ophthalmologists and a lack of funds for screening. Almost all countries lacked oxygen delivery systems and oxygen saturation monitors. All countries fear an epidemic of blind infants as a result of ROP. In our view, the results of our survey indicate that it is possible to reduce the incidence of ROP, also in LMIC. The first step to stop this epidemic is to be aware of the risks of ROP. This concerns all those involved in the care of preterm infants, paediatricians, ophthalmologists, nurses and administrators.

Confidential: For Review Only

Our study found that the screening rate for ROP is rather low in Indonesia, both in infants with a low gestational age and in infants with a higher gestational age who received supplemental oxygen for a prolonged period. This is most likely due to at least three factors: a lack of trained ophthalmologists, a lack of awareness among paediatricians of the importance of screening and a lack of funding for ophthalmologists. Paediatric ophthalmologists are mainly found in large academic hospitals and the national centres for perinatology. In almost all cases, there is only one paediatric ophthalmologist who is not always available. In order to increase the screening rate, paediatricians must be made aware of its importance and ophthalmologists must be trained to do it. Funds to carry out the screening need to be made available.

We found no difference in the incidence of ROP in the institutions with a screening rate of at least 80%, compared to the whole group of infants. This indicates that a high number of infants are not screened while they develop ROP. The follow-up of preterm infants admitted to a NICU is low in Indonesia because of socioeconomic factors. It is not therefore known how often ROP was present in surviving, unscreened infants.

A lack of ophthalmologists trained in ROP screening is not a problem that is unique to Indonesia. There is a shortage of trained ophthalmologists in many LMIC and, where they do exist, screening is often not properly reimbursed and only the most committed ophthalmologists are willing to screen.[23]

In addition, paediatricians participating in this survey reported a reluctance among ophthalmologists to screen because of medico-legal problems, an imbalance between the level of difficulty and time spent examining very small premature babies and the results obtained, and the asynchronous examination fees in different health insurance systems. This

Confidential: For Review Only

all contrasts with the practice in HIC, where failure to screen an eligible infant for ROP could be considered malpractice.

The incidence of ROP in Harapan Kita Women and Children's Hospital was significantly lower in the period 2016-2017 than in the period 2005-2015. At the same time, the screening rate improved to 93%. This indicates that ROP declined significantly at that hospital. In 2016, a strict policy was introduced to set the oxygen saturation monitor at 91% to 95% for preterm infants. The use of oxygen in the delivery room was also strictly regulated, with resuscitation of preterm infants starting at 30% oxygen. CPAP was given directly after birth and continued in the NICU. These measures are likely to have played an important role in reducing the incidence of ROP at that NICU.

The highest rate of both mortality and ROP was found in the university-based hospitals. These are referral hospitals that admit mothers with antenatal complications. They may therefore treat the sickest infants, sicker than those cared for in governmental and private hospitals. This could explain the higher rate of mortality and ROP. At the same time, however, the two national centres are also referral hospitals and they showed a lower mortality rate and a lower incidence of ROP (figures 1,2). This suggests that there could be other factors behind the higher incidence of ROP in the university-based hospitals. More liberal use of oxygen might be a cause. More studies are needed to determine the cause of the less favourable outcome in the university-based hospitals.

A limitation of this study is that the survey on the incidence of ROP as carried out in this study had not been done before. As a reference in this paper, we used data from one referral hospital as well as data from the literature. The rates of ROP found in one hospital in Indonesia[1] and the studies done in other centres[2] all showed a high incidence of ROP in the

Confidential: For Review Only

period before 2015. Therefore, we are convinced that the incidence of ROP was indeed much higher than suggested by the results of this survey. A second limitation of our survey is that we only included inborn infants. We do not have precise data on the ratio of inborn and outborn infants for all hospitals. In the Harapan Kita Hospital, one of the national referral hospitals, on average 71% of the admitted infants are inborn. We estimate that this percentage will be almost the same for the university-based NICUs. Unfortunately, there is no adequate neonatal transport service in Indonesia. Transportation is carried out by poorly trained personnel and only 100% oxygen can be given during transport. The referring and accepting neonatologists do not meet, making the transfer of information difficult and often incomplete.

Conclusion

The incidence of ROP in preterm infants in Indonesia was lower in 2016-2017 than in the period before 2015. This may be due to a higher awareness of ROP among practitioners and the introduction of a national healthcare plan. Our data, however, are likely to be an underestimation of the real incidence of ROP because of the higher mortality rate among small premature infants in Indonesia than in HIC and a low rate of screening and follow-up of surviving preterm infants.

Acknowledgements The authors would like to thank all infants and families whose data contributed to the IMSROP Study Group. We wish to thank our ophthalmologists Dr Habsyiyah, Dr Nani H. Widodo and Dr Florence Manurung. Together with all the ophthalmologists at each hospital included in this study, they were involved in examining patients and providing scientific input in the field of ophthalmology. We would also like to

Confidential: For Review Only

thank Prof. Rita S. Sitorus, who encouraged us to work together to reduce the incidence of ROP in Indonesia in terms of neonatology, ophthalmology and public health.

Collaborators

*Members of the IMSROP (Indonesian Multicenter Retinopathy of Prematurity) Study Group: Rinawati Rohsiswatmo *(Rumah Sakit Cipto Mangunkusumo, Jakarta Pusat)*, Tetty Yuniati *(RSUP Hasan Sadikin, Bandung, Jawa Barat)*, Gatot Irawan Sarosa *(RSUP Dr Kariadi, Semarang, Jawa Tengah)*, Dwi Hidayah *(RSUD Dr Moewardi, Solo, Jawa Tengah),* Dina Angelika dan Risa Etika *(RSUD Dr Soetomo Surabaya, Jawa Timur)*, Eko Sulistijono *(RSUD Dr Syaiful Anwar Malang, Jawa Timur)*, Made Kardana *(RSUP Sanglah, Denpasar, Bali)*, Pertin Sianturi *(RSUP H. Adam Malik, Medan, Sumatera Utara)*, Eni Yantri *(RSUP Dr M. Djamil Padang, Sumatera Barat)*, Dewi Anggraini Wisnumurti *(RSUD Arifin Achmad Pekanbaru, Riau)*, Afifa Ramadanti *(RSUP M. Hoesin Palembang, Sumatera Selatan)*, Pudji Andayani *(RSUD Ulin Banjarmasin Kalimantan Selatan)*, Rocky Wilar *(RSUP Prof. Dr RD Kandou Hospital, Manado)*, Ema Alasiry (RSUP Dr Wahidin Sudirohusodo, Makasar, Sulawesi Selatan), Harris Alfan *(RS Atma Jaya, Jakarta Utara)*, Johanes Edy Siswanto *(RSAB Harapan kita dan RS Royal Taruma, Jakarta Barat)*, Naomi Esthernita *(Siloam Hospital Kebon Jeruk, Jakarta Barat)*, Fahrul Wakil Arbi *(RS Budi Kemuliaan, Jakarta Pusat)*, Nita Dewanti *(RS Premier Bintaro, Jakarta Selatan)*, Agnes Yuni Purwita Sari *(RSUP Persahabatan, Jakarta Timur)*, Ellen Sianipar *(RSUD Pasar Rebo Jakarta Timur)*, Robert Soetandio *(RS Awal Bros Hospital, Tangerang, Jawa Barat)*, Andika Tiurmaida *(RSUD Cibinong, Jawa Barat)*, Thomas Harry Adoe *(RSUD Kota Bekasi)*, Muda Isa Ariantana *(Santosa Hospital Bandung Cermai Bandung, Jawa Barat)*, Samad *(RSUP Dr Soeradji Tirtonegoro Klaten),* Anince *(RS Bakti Timah Karimun, Kepulauan Riau)*, Tri Putri Yuliani *(RSUD Siti Aisyah Kota Lubuk Linggau)*, Sumsel, Dina Frida *(RSUD Dr Sudarso, Kalimantan Barat dan RS Anugrah Bunda Khatulistiwa, Kalimantan Barat)*, Nurhandini Eka Dewi *(RSUD Mataram, Nusa Tenggara Barat)*, Woro Indri P. Purba *(RSUD Prof. Dr WZ Johannes Kupang, Nusa Tenggara Timur),* Sadiyah *(RSUD I Lagaligo, Luwu Timur, Sulawesi Selatan)*.

Contributors JES is the person directly involved in all aspects of this research and manuscript. JES, AB, PD and PJJS contributed to the study design and analysis. PJJS assisted in the literature review and wrote the report and the discussion section. AFB and PJJS are the guarantors for the study. JES, AB and PJJS had full access to the IMSROP data. All authors had full access to the summary data presented in this paper (including statistical reports and tables) and take responsibility for the integrity of the data and the accuracy of the data analysis.

Confidential: For Review Only

Funding The authors have not declared a specific grant for this research from any funding agency in the public, commercial or not-for-profit sectors.

Conflict of interests None declared.

Patient consent Detail has been removed from these case descriptions to ensure anonymity. The editors and reviewers have seen the detailed information available and are satisfied that the information backs up the case the authors are making.

Ethics approval The study was approved by the Institutional Review Board of the University of Indonesia School of Public Health (No. 32/H2.F10/PPM.00/13).

Provenance and peer review Not commissioned; externally peer-reviewed.

Confidential: For Review Only

What is already known on this topic?

► Several studies in the previous decade in Indonesia showed a high incidence of ROP, which is a serious problem, as in other LMIC countries.

► The high rate of ROP in Indonesia is likely due to the expansion of neonatal intensive care, whereas there is a lack of awareness of the risks of developing ROP.

What does this study add?

► The incidence of ROP in Indonesia was lower in 2016-2017 than in surveys conducted before 2015.

► There was a difference in mortality and rate of ROP between the types of hospital. Both mortality and the incidence of ROP were highest in university-based hospitals.

► The lower rate of ROP could be the result of a new national guideline on ROP, an

increased awareness following meetings and the introduction of national health care.

References

1. Siswanto JE, Nani H Widodo NH, Sauer PJJ. *Arch Dis Child 2018*;103:619–21.
2. Siswanto JE, Sauer PJ. Retinopathy of prematurity in Indonesia: Incidence and riskfactors. J *Neonatal Perinatal Med* 2017;10:85–90.
3. Sitorus RS et al. POKJA Nasional ROP dan Bayi Prematur, Pedoman Nasional: Skrining dan Terapi ROP pada bayi prematur di Indonesia. eds 1. 2010
4. Gilbert C, Blencowe H. Retinopathy of prematurity: it is time to take action. Comm Eye Health Vol. 30 No. 99 2017 pp 45-48
5. Darlow BA, Gilbert CE, Quirogaet AM. Setting up and improving retinopathy of prematurity pros: interaction of neonatology, nursing and ophthalmology. Clin Perinatol 2013;40:215–27
6. Mataftsi A, Moutzouri S, Karagianni P, et al. Retinopathy of prematurity occurrence and evaluation of screening policy in a large tertiary Greek cohort. *Int Ophthalmol* 2020;40:385-91
7. Austeng D, Källen K, Hellström A, et al. Regional differences in screening for retinopathy of prematurity in infants born before 27 weeks of gestation in Sweden – the EXPRESS study. *Acta Ophthalmol* 2014; 92:311-5.
8. Draper ES, Zeitlin J, Field DJ, et al. Mortality patterns among very preterm babies: A comparative analysis of two European regions in France and England. *Arch Dis Child Fetal Neonatal Ed* 2007; 92: 356–360.
9. Milligan DW. Outcomes of children born very preterm in Europe. *Arch Dis Child Fetal Neonatal Ed* 2010;95:F234-40.
10. Draper ES, Zeitlin J, Fenton AC, et al, on behalf of the MOSAIC research group. Investigating the variations in survival rates for very preterm infants in 10 European regions: the MOSAIC birth cohort. *Arch Dis Child Fetal Neonatal Ed* 2009;3:F158-63.
11. Mohangoo AD, Buitendijk SE, Szamotulska K, et al. Gestational Age Patterns of Fetal and Neonatal Mortality in Europe: Results from the Euro-Peristat Project. *PLoS ONE* 2011;6:e24727.
12. Serenius F, Sjörs G, Blennow M, et al, and For the EXPRESS study group*. EXPRESS study shows significant regional differences in 1-year outcome of extremely preterm infants in Sweden. *Acta Paediatr* 2014;103:27–37.
13. Santhakumaran S, Statnikov Y, Gray D, et al, on behalf of the Medicines for Neonates Investigator Group. Survival of very preterm infants admitted to neonatal care in England 2008–2014: time trends and regional variation. *Arch Dis Child Fetal Neonatal Ed* 2018;103:F208–F215.

Confidential: For Review Only

14. Norman M, Hallberg B, Abrahamsson T, et al. Association Between Year of Birth and 1-Year Survival Among Extremely Preterm Infants in Sweden During 2004-2007 and 2014-2016. *JAMA* 2019; 321:1188-1199.
15. Lee SK, McMillan DD, Ohlsson A, et al. Variations in practice and outcomes in the Canadian NICU network: 1996-1997. *Pediatrics* 2000;106:1070–9.
16. Stoll BJ, Hansen NI, Bell EF, et al. Neonatal Outcomes of Extremely Preterm Infants From the NICHD Neonatal Research Network. *Pediatrics* 2010; 126: 443–456.
17. Stoll BJ, Hansen NI, Bell EF, et al. Trends in Care Practices, Morbidity, and Mortality of Extremely Preterm Neonates, 1993-2012. *JAMA* 2015;314:1039-51.
18. Su BH, Hsieh WS, Hsu CH, et al. Premature Baby Foundation of Taiwan (PBFT). Neonatal Outcomes of Extremely Preterm Infants From Taiwan: Comparison With Canada, Japan, and the USA. *Pediatr Neonatol* 2015;56:46-52.
19. Corpus K, Jimenez IV JM, Anzures R, Bascuna RI, Ventura R, Reandelar Jr M. Retinopathy of Prematurity Working Group. Proposed New Retinopathy of Prematurity Screening Criteria: Evidence for Including Older and Heavier Filipino Premature Babies. Philipp J Ophthalmol 2013;38:72-79.
20. Ali NAM, George J, Joshi N, et al. Prevalence of retinopathy of prematurity in Brunei Darussalam. Int J Ophthalmol 2013;3: 381–384.
21. Trinavarat A, Atchaneeyasakul L, Udompunturuk S. Applicability of American and British criteria for screening of retinopathy of prematurity in Thailand. Jpn J Ophthalmol 2004;48:50-3.
22. Bowe T, Nyamai L, Ademola-Popoola D, et al. The current state of retinopathy of prematurity in India, Kenya, Mexico, Nigeria, Philippines, Romania, Thailand, and Venezuela. Digit J Ophthalmol 2019;4: 49–58.
23. Gilbert CE. Screening for retinopathy of prematurity: does one size fit all? Arch Dis Child Fetal Neonatal Ed July 2016 Vol 101 No 4

Figure legend

Figure 1. The incidence of ROP based on gestational age in each group of hospitals in Indonesia 2016-2017.

Figure 2. Mortality based on the gestational age in each group of hospitals in Indonesia 2016-2017

Table 1. Hospital-based ROP data surveillance in Indonesia 2016-2017 (GA ≤ 34 weeks)

Type of hospital	Total hospitals	Total infants	Survived	Died		Screened		ROP	
				n	%	n	%	n	%
RSCM/NRH	1	1038	890	148	14.3	392	44.0	7	1.8
HKWCHC	1	478	392	86	18.0	281	71.7	7	2.5
UBH	14	6549	4738	1811	27.7	2041	43.1	197	9.7
OH	18	4050	3261	789	19.5	711	21.8	17	2.4
- Government hospital	10	3020	2425	595	19.7	605	24.9	15	2.5
- Private hospital	8	1030	836	194	18.8	106	12.7	2	1.9
All hospitals	34	12115	9281	2834	23.4	3425	36.9	228	6.7

Note: RSCM/NRH: Ciptomangunkusumo Hospital (national referral hospital); HKWCH: Harapan Kita Women and Children's Hospital (National Centre for Women and Children's Health); UBH: university-based hospital; OH: other hospital; All: all hospitals; ROP; retinopathy of prematurity.

Confidential: For Review Only

Table 2a. ROP incidence in Harapan Kita Women and Children's Hospital, Indonesia based on the gestational age in 2005-2017

Variable		Infants GA <28 wks		Infants GA 28 - 32 wks		Infants GA >32 - 34 wks	
		2005-2015	2016-2017	2005-2015	2016-2017	2005-2015	2016-2017
Total (inborn infants)	n	185	54	569	221	NA	203
Died	n	91	27	126	24	-	24
	%	49	50	22	11	-	12
Survived	n	94	27	443	197	-	179
Screened	n	47	25	261	169	156	87
Screened/Survived	%	50	93	59	86	-	49
No ROP	n	28	23	187	167	126	84
ROP 1-2	n	9	2	66	1	29	3
ROP 3-5	n	10	0	8	1	1	0
Prevalence of Any ROP	%	40	8	28	1	19	3
Prevalence of Severe ROP	%	21	0	3	1	1	0

Note: ROP: Retinopathy of Prematurity; GA: gestational age; wks: weeks.

Confidential: For Review Only

Table 2b. ROP incidence in Indonesia based on the gestational age in 2016-2017

Variable		Infants GA <28 weeks					Infants GA 28 - 32 weeks					Infants GA >32-34 weeks				
		2016-2017					2016-2017					2016-2017				
		RSCM	HKWCH	UBH	OH	All	RSCM	HKWCH	UBH	OH	All	RSCM	HKWCH	UBH	OH	All
Total (inborn infants)	n	115	54	903	354	1426	501	221	2288	2044	5054	422	203	3358	1652	5635
Died	n	87	27	577	259	961	122	24	700	349	1195	26	24	534	181	765
	%	76	50	64	73	67	24	11	31	17	24	6	12	16	11	14
Survived	n	28	27	326	95	465	379	197	1588	1695	3859	396	179	2824	1471	4870
Screened	n	23	25	204	40	292	187	169	923	315	1594	182	87	914	356	1539
Screened/Survived	%	82	93	63	42	63	49	86	58	19	41	46	49	32	24	32
No ROP	n	22	23	161	33	239	185	167	751	309	1478	178	84	441	352	1480
ROP 1-2	n	1	2	34	5	42	2	1	94	6	103	2	3	45	2	52
ROP 3-5	n	0	0	9	2	11	0	1	12	0	13	2	0	3	2	7
Prevalence of Any ROP	%	4	8	21	18	18	1	1	11	2	7	2	3	5	1	3.8
Prevalence of Severe ROP	%	0	0	4	5	4	0	1	1	0	1	1	0	0	1	0.4

Note: RSCM/NRH: Ciptomangunkusumo Hospital (national referral hospital); HKWCH: Harapan Kita Women and Children Hospital (National Centre for Women and Children's Health); UBH: university-based hospital; OH: other hospital; All: all hospitals; ROP; Retinopathy of Prematurity; GA: gestational age.

Confidential: For Review Only

Table 3. Incidence of ROP in hospitals with 80% screening coverage in Indonesia, 2016-2017

Variable		Birth weight					Gestational age				
		<1000 g	1000-1500 g	>1500-2000 g	>2000-<2500 g	≥2500 g	<28 wks	28-32 wks	32-34 wks	34-<37 wks	≥37 wks
Total (inborn)	n	585	1541	2679	4522	20572	560	2144	2319	3913	20963
Died	n	394	449	303	163	266	391	508	244	165	267
	%	67	29	11	4	1	70	24	11	4	1
Survived	n	191	1092	2376	4359	20306	169	1636	2075	3748	20696
Screened	n	172	1040	1033	1186	14	168	1147	926	1199	5
Screened/Survived	%	90	95	43	27	0	99	70	45	32	0
No ROP	n	137	966	978	1159	14	132	1074	874	1169	5
ROP 1-2	n	28	57	53	27	0	27	62	46	30	0
ROP 3-5	n	7	17	2	0	0	9	11	6	0	0
Incidence of Any ROP	%	20	7	5	2	0	21	6	6	3	0
Incidence of Severe ROP	%	4	2	0	0	0	5	1	1	0	0

Note: these data come from 13 of 34 hospitals; ROP: retinopathy of prematurity

Confidential: For Review Only

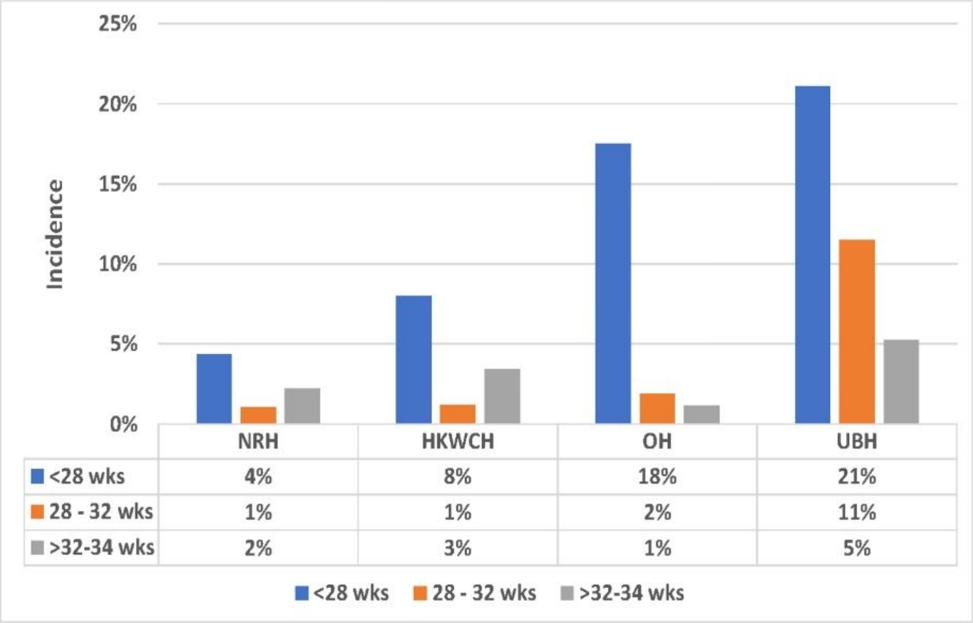

Note: NRH/RSCM: National Referral Hospital (Ciptomangunkusumo Hospital; HKWCH: Harapan Kita Women and Children Hospital (National Centre for Women and Children's Health); UBH: University Based Hospital; OH: Other Hospital; All: All Hospital; wks: weeks.

248x180mm (96 x 96 DPI)

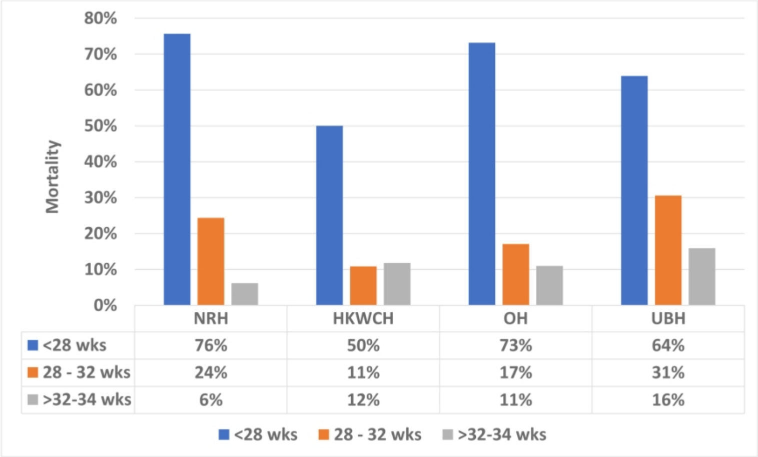

Note: RSCM/NRH: Ciptomangunkusumo Hospital (National Referral Hospital); HKWCH: Harapan Kita Women and Children Hospital (Health Centre); UBH: University Based Hospital; OH: Other Hospital.

148x105mm (600 x 600 DPI)

Supplementary Table. Incidence of ROP in Indonesia based on the birth weight in 2005-2017

Variable		Inborn infants BW <1000 gram						Inborn infants BW 1000-1500 gram						Inborn infants BW >1500-2000 gram					
		2005-2015	2016-2017					2005-2015	2016-2017					2005-2015	2016-2017				
		HKWCH	RSCM	HKWCH	UBH	OH	All Hospital	HKWCH	RSCM	HKWCH	UBH	OH	All Hospital	HKWCH	RSCM	HKWCH	UBH	OH	All Hospital
Total (Inborn)	n	182	132	71	1024	361	1588	437	306	160	2104	1094	3664	748	475	236	3730	2055	6496
Died	n	85	101	38	567	256	962	72	93	24	640	346	1103	.	42	24	532	205	803
	%	47	77	54	55	71	61	16	30	15	30	32	30	.	9	10	14	10	12
Survived	n	97	31	33	457	105	626	365	213	136	1464	748	2561	.	433	212	3198	1850	5693
Screened	n	59	26	29	197	35	287	252	169	123	802	273	1413	163	177	54	1131	495	1857
Screened/Survived	%	61	84	88	43	33	46	69	79	90	55	36	55	.	41	25	35	27	33
No ROP	n	27	24	26	156	28	234	172	167	120	696	265	1248	139	175	53	488	492	1208
ROP 1-2	n	22	2	2	33	5	42	71	1	3	89	7	100	22	1	1	54	2	58
ROP 3-5	n	10	0	1	6	2	9	9	1	0	17	1	19	2	1	0	0	1	2
Prevalence of Any ROP	%	54	8	10	20	20	18	32	1	2	13	3	8	15	1	2	5	1	3
Prevalence of Severe ROP	%	17	0	3	3	6	3	4	1	0	2	0	1	1	1	0	0	0	0

Note: RSCM/NRH: Ciptomangunkusumo Hospital (National Referral Hospital); HKWCH: Harapan Kita Women and Children Hospital (National Centre for Women and Children's Health); UBH: University Based Hospital; OH: Other Hospital; All: All Hospital; ROP; Retinopathy of Prematurity; BW: Birth weight.

Confidential: For Review Only

BMJ Paediatrics Open

A multicentre survey of retinopathy of prematurity in Indonesia

Journal:	*BMJ Paediatrics Open*
Manuscript ID	bmjpo-2020-000761.R2
Article Type:	Original research
Date Submitted by the Author:	20-Sep-2020
Complete List of Authors:	Siswanto, J; Rumah Sakit Anak dan Bunda Harapan Kita, Dep. Neonatology; Pelita Harapan University Faculty of Medicine, Pediatric<br>Bos, Arend F.; University Medical Center Groningen Intensive Care Medicine, Neonatology<br>Dijk, Peter; Universitair Medisch Centrum Groningen, Neonatology<br>Rohsiswatmo, Rinawati ; University of Indonesia Faculty of Medicine, Pediatric<br>Irawan, Gatot; Dr Kariadi General Hospital Medical Center, Pediatric<br>Sulistijono, Eko; Dr Saiful Anwar General Hospital, Pediatric<br>Sianturi, Pertin; University of Sumatera Utara Faculty of Medicine, Pediatric<br>Wisnumurti, Dewi; University of Riau Faculty of Medicine, Pediatric<br>Wilar, Rocky; Sam Ratulangi University Faculty of Medicine, Pediatric<br>Sauer, Pieter; Universitair Medisch Centrum Groningen, Neonatology<br>Study Group, The IMSROP
Keywords:	Neonatology, Ophthalmology, Epidemiology, Mortality, Health services research

SCHOLARONE™
Manuscripts

I, the Submitting Author has the right to grant and does grant on behalf of all authors of the Work (as defined in the below author licence), an exclusive licence and/or a non-exclusive licence for contributions from authors who are: i) UK Crown employees; ii) where BMJ has agreed a CC-BY licence shall apply, and/or iii) in accordance with the terms applicable for US Federal Government officers or employees acting as part of their official duties; on a worldwide, perpetual, irrevocable, royalty-free basis to BMJ Publishing Group Ltd ("BMJ") its licensees and where the relevant Journal is co-owned by BMJ to the co-owners of the Journal, to publish the Work in this journal and any other BMJ products and to exploit all rights, as set out in our licence.

The Submitting Author accepts and understands that any supply made under these terms is made by BMJ to the Submitting Author unless you are acting as an employee on behalf of your employer or a postgraduate student of an affiliated institution which is paying any applicable article publishing charge ("APC") for Open Access articles. Where the Submitting Author wishes to make the Work available on an Open Access basis (and intends to pay the relevant APC), the terms of reuse of such Open Access shall be governed by a Creative Commons licence – details of these licences and which Creative Commons licence will apply to this Work are set out in our licence referred to above.

Other than as permitted in any relevant BMJ Author's Self Archiving Policies, I confirm this Work has not been accepted for publication elsewhere, is not being considered for publication elsewhere and does not duplicate material already published. I confirm all authors consent to publication of this Work and authorise the granting of this licence.

Confidential: For Review Only

Title: A multicentre survey of retinopathy of prematurity in Indonesia

First Author/Corresponding Author: **J. Edy Siswanto**

Corresponding Author's Institution:

Department of Neonatology, Harapan Kita Women and Children's Hospital

Postal Address:

Rumah Sakit Anak dan Bunda Harapan Kita

Jl. Letjen S. Parman Kav. 87

Jakarta Barat, DKI Jakarta, ID 11420

Indonesia

Email: edyjo15@yahoo.com, edy.jos@gmail.com

Telephone number: +62215668284 and +6287786293779

Fax number: +62215657547

Keywords: Retinopathy of Prematurity, incidence, mortality

Word count: ~~2239~~

Running title: Incidence of ROP, mortality, Indonesia

Confidential: For Review Only

A multicentre survey of retinopathy of prematurity in Indonesia

J. Edy Siswanto[1a, b], Arend F. Bos[2],
Peter Dijk[2], Rinawati Rohsiswatmo[3], Gatot Irawan[4], Eko Sulistijono[5], Pertin Sianturi[6],
Dewi A. Wisnumurti[7], Rocky Wilar[8],
Pieter JJ Sauer[2], for the IMSROP study group*

Author information

[1a]Neonatology Working Group, Department of Pediatrics, Harapan Kita Women and Children's Hospital, Jakarta, Indonesia

[1b]Faculty of Medicine, Pelita Harapan University, Tangerang

[2]Department of Pediatrics, Beatrix Children's Hospital, University Medical Center Groningen, Groningen, The Netherlands

[3]Department of Pediatrics, Faculty of Medicine, University of Indonesia, Rumah Sakit Cipto Mangunkusumo, Jakarta

[4]Department of Pediatrics, Faculty of Medicine, Diponegoro University, RSUP Dr Kariadi, Semarang, Central Java

[5]Department of Pediatrics, RSUD Dr Syaiful Anwar, Faculty of Medicine, Brawijaya University Malang, East Jawa

[6]Department of Pediatrics, Faculty of Medicine, University Sumatera Utara (USU), RSUP H. Adam Malik, Medan, North Sumatera

[7]Department of Pediatrics, RSUD Arifin Achmad Pekanbaru, Faculty of Medicine, Riau University

[8]Department of Pediatrics, RSUP Prof. Dr RD Kandou Hospital, Faculty of Medicine, Sam Ratulangi University, Manado, North Sulawesi

Abstract

Background The incidence of retinopathy of prematurity (ROP) is higher in Indonesia than in high-income countries. In order to reduce the incidence of the disease, a protocol on preventing, screening, and treating ROP was published in Indonesia in 2010. To assist the practical implementation of the protocol, meetings were held in all Indonesia regions, calling attention to the high incidence of ROP and the methods to reduce it. In addition, national health insurance was introduced in 2014, making ROP screening and treatment accessible to more infants.

Confidential: For Review Only

Objective To evaluate whether the introduction of both the guideline drawing attention to the high incidence of ROP and national health insurance may have influenced the incidence of the disease in Indonesia.

Setting Data were collected from 34 hospitals with different levels of care: national referral centers, university-based hospitals, and public and private hospitals.

Methods: A survey was administered with questions on admission numbers, mortality rates, ROP incidence, and its stages for 2016-2017 in relation to gestational age and birth weight.

Results We identified 12,115 eligible infants with a gestational age of less than 34 weeks. Mortality was 24% and any-stage ROP 6.7%. The mortality in infants aged less than 28 weeks was 67%, the incidence of all-stage ROP 18%, and severe ROP 4%. In the 28-32 week group, the mortality was 24%, all-stage ROP 7%, and severe ROP 4-5%. Both mortality and the incidence of ROP were highest in university-based hospitals.

Conclusions In the 2016-2017 period, the infant mortality rate before 32 weeks of age was higher in Indonesia than in high-income countries, but the incidence of ROP was comparable. This incidence is likely an underestimation due to the high mortality rate. The ROP incidence in 2016-2017 is lower than in surveys conducted before 2015. This decline is likely due to a higher practitioner awareness about ROP and national health insurance implementation in Indonesia.

Introduction

Studies on the incidence of retinopathy of prematurity (ROP) during the period 2005-2015 in Indonesia showed a relatively high incidence of ROP.[1, 2] ROP was also seen in infants with gestational ages above 32 weeks. In view of these findings, a group of neonatologists and ophthalmologists met in 2009 and 2010 to develop a guideline for the prevention, early

Confidential: For Review Only

detection and management of ROP for neonatologists and ophthalmologists in Indonesia. The national guideline, which was published in 2010, provides advice on how to prevent ROP and how and when to screen newborn infants.[3] It recommends screening all infants born before 34 weeks or with a birthweight of less than 1500 g, as well as infants of higher gestational age and higher birth weight who received oxygen for a prolonged period. Neonatologists and paediatric ophthalmologists realized that the guideline's publication had almost no impact on clinical practice. This was most likely due to a lack of knowledge about ROP among paediatricians and ophthalmologists and to financial constraints in hospitals.

After 2010, the paediatric and ophthalmologist societies in Indonesia therefore organized several meetings to alert their members to the high incidence of ROP and to ways to prevent the disease and perform screening procedures. In addition, national health insurance was introduced in 2014, thereby increasing opportunities to treat premature infants and to improve ROP screening. Care for preterm infants in Indonesia is provided by two national referral centres, university-based NICUs (neonatal intensive care centres), as well as governmental and private hospitals. There are marked variations among these hospitals, such as patients' socioeconomic and cultural backgrounds and other demographic factors, in terms of their options for caring for sick preterm infants. We do not know whether the differences between hospitals resulted in a different incidence of ROP.

To evaluate whether an awareness of the high incidence of ROP and the introduction of national health insurance may have reduced that incidence, we conducted a survey on the incidence of the disease at Indonesian neonatal intensive care centres and local hospitals in the years 2016-2017. As the incidence may differ between hospitals, we collected data from all levels of hospitals that provide care for sick newborns.

Confidential: For Review Only

Methods

This is a survey: we collected data for the years 2016-2017 in the period from March to November 2019. Paediatricians in 47 hospitals were contacted by email and direct phone calls; 41 were willing to send us the required information. We received responses from 34 hospitals in 17 major provinces of Indonesia – 16 teaching hospitals, two of which are national referral hospitals for perinatology, and 10 government and eight private hospitals. The availability of NICU beds varied greatly across between regions because of a lack of trained neonatologists and differences in stakeholder support in the province or district where the paediatricians worked. We approached hospitals offering all levels of neonatal care, located in all the different parts of Indonesia.

The survey asked for the following data: number of inborn preterm infants, number of preterm infants who died in the perinatal period, number of infants screened for ROP, number of infants with ROP and the stage of ROP. For the sake of uniformity and to analyse all the data, we asked for inborn babies to be further categorised by gestational age and birth weight. For ROP, we used the terms mild ROP (stages 1-2) and severe ROP (stage 3 or higher). We included only inborn infants because important data such as gestational age and complications in pregnancy are often not available for outborn infants. We analysed the university-based hospitals and the district (government)/private hospitals in two separate groups. The data for the two national referral perinatal centres are shown separately. The results are described in frequencies and percentages. No advance statistical test was used for the data analysis.

Patient involvement

Confidential: For Review Only

Neither patients nor the public were involved in the design, execution, reporting or dissemination plans for our research.

Results

We received data from the two national referral hospitals for perinatology (Harapan Kita Women and Children's Health Centre and RSCM-Ciptomangunkusumo Hospital), 14 university-based hospital NICUs and 18 other hospitals, which included 10 government hospitals and eight private hospitals (Table 1).

In total, we received data on 12,115 infants with a gestational age <34 weeks, 5,252 of whom had a birth weight of less than 1500 g. The overall mortality of infants <34 weeks was 24.1%. Almost 37% of surviving infants were screened for ROP; the incidence of all-stage ROP was 6.7%. The highest incidence of both mortality and ROP was found the university-based hospitals (Table 1).

Table 2 shows the data for each hospital or group of hospitals according to gestational age. The overall mortality in the group <28 weeks was 67%, with no differences between hospitals. Sixty-three percent of surviving infants were screened for ROP. The rate of screening ranged from 93% in one referral hospital to 42% in the 'other hospitals' group. The overall rate of any-stage ROP was 18% and severe ROP 4%. The incidence of both any-stage ROP and severe ROP was higher in the university-based NICUs and other hospitals than in the two national centres (18 and 21% vs 4 and 8%) (Figure 1). Severe ROP was not seen in the national centres, but was found in 4% and 5% of infants in the university and other hospitals respectively. The same trend was found in infants born after 28-32 weeks. Overall mortality in this group was 24%, with the highest mortality in the university hospitals (31%), compared with 11% in one of the national centres. The rate of screening in the 28-32 week group was lower than in the

Confidential: For Review Only

<28 week group (41 vs 63%) and ranged from 86% in one national centre to 19% in the 'other hospitals' group. Any stage ROP was found in 7% of surviving infants, with the highest incidence (11%) in university hospitals and 1-2% in other hospitals. The incidence of severe ROP was 0-1% in all hospitals. The mortality in the 32-34 week group was 14% on average, with no important differences between hospitals. The rate of screening in these infants was 32% and the prevalence of any stage ROP was 4%, with the highest incidence in university-based hospitals.

Confide

The supplementary table shows the data for each hospital or group of hospitals according to birth weight. The overall mortality in the <1000 g group was 61%, with no differences between groups of hospitals. Forty-six percent of surviving infants were screened for ROP; the rate of screening ranged from 88% in one referral hospital to 33% in the 'other hospitals' group. The overall rate of any stage ROP was 18% and severe ROP 3%. The incidence of both any stage ROP and severe ROP was higher in the university-based NICUs and other hospitals than in the two national centres (20% vs 8 and 10%). Severe ROP was not encountered in the national centres but was seen in 3% and 6% respectively of infants in the university and other hospitals. The same trend was found in infants born with a birth weight of 1000-1500 g. Overall mortality in this group was 30%, with the highest mortality in the university and other hospitals (30%), compared with 15% in one of the national centres. The rate of screening in the 1000-1500 g group was higher than in the <1000 g group (55% vs 46%), and ranged from 90% in one national centre to 36% in the 'other hospitals' group. Any-stage ROP was found in 8% of surviving infants, with the highest incidence (13%) in the university hospitals and 3% in other hospitals. The incidence of severe ROP was 1% in all hospitals. The mortality in the >1500-2000 g group was 12% on average, with no significant differences between hospitals.

The rate of screening in these infants was 33% and the prevalence of any stage ROP was 3%, with the highest incidence in university-based hospitals (5%).

In order to assess whether the screening rate may have affected the incidence of ROP found in our survey, we calculated the incidence of ROP in 13 of the 34 hospitals where at least 80% of infants were screened once or more (table 3). There was no difference in the rate of ROP between these hospitals and the total group. In the <28 weeks group, the ROP rate at all stages was 21% and 5% for severe ROP. In the 28-32 weeks group, the incidence of all ROP stages was 6% and severe ROP 1%.

Discussion

Globally, ROP is one of the hidden causes of morbidity following death-related problems such as respiratory distress (asphyxia), infections and other complications that arise in small, sick preterm infants. Countries with a high rate of premature births will consequently see a higher number of infants affected by ROP. This situation occurs in almost all parts of the world, both in high-income (HIC) and lower middle income countries (LMIC).[4]

What has been characterised as the third ROP epidemic has particularly affected countries in Southeast Asia. It is clear that many cases of visual impairment due to ROP are preventable through improved neonatal care, timely retinal examination and appropriate treatment.[5] In this survey, we present data on the incidence and severity of ROP following the implementation of a national guideline for the prevention, screening and treatment of ROP and the introduction of national health insurance in Indonesia, one of the major regions in Southeast Asia.

Confidential: For Review Only

This survey conducted in 2016-2017 found the incidence of ROP in Indonesia in that period to be much lower than the incidence we found in 2005-2015.[1,2] In that previous period, we found in one NICU a 40% incidence of all-stage ROP in infants below 28 weeks and a 28% incidence in the 28-32 week group.[1] Other studies from the period 2005-2015 in Indonesia found a ROP incidence of 18-30% in infants born before 32 weeks and/or with a birth weight of less than 1500 g.[2] Different factors may have contributed to this marked decline in ROP. Firstly, a national guideline for ROP prevention and screening was published in 2010. When we realized that simply publishing this guideline did not change practices in Indonesia, several focus meetings were held across the country to alert practitioners to the high incidence of ROP, the methods to prevent ROP and the importance of screening.[3] In addition, national health insurance was introduced in 2014, thereby improving opportunities for treating and screening preterm infants. We cannot identify which of these factors may have had the greatest impact on the decline in incidence of ROP.

It is difficult to compare the incidence of ROP in Indonesia with the incidence reported in HIC. A recent study from Greece showed an incidence of any stage ROP in infants <32 weeks of 19.7% and severe ROP 7.4%.[6] The EXPRESS study from Sweden found an incidence of any stage ROP in infants <31 weeks of 31.9% and severe ROP 5.7%.[7] There is a marked difference, however, between Indonesia and HIC in the type of infants cared for and their survival. In Indonesia, almost no infants born after a gestational age of 26 weeks or less will survive. Mortality among infants of 26-28 weeks is much higher than in HIC. In our survey, we found that 67% of infants born <28 weeks died. Recent data from Sweden, England, France, the Netherlands, Canada and the USA show a survival rate for infants born between 26-28 weeks of 80-90%.[8-18] In Indonesia, only the healthier very preterm infants will survive. The incidence of ROP may be lower in these infants than in the very sick newborns who died. The same may

Confidential: For Review Only

be true of infants born after 28-32 weeks. In Indonesia, we found that 24% of these infants died. In HIC, that figure is less than 5%. The present data on the incidence of ROP in Indonesia could be an underestimation caused by the higher death rate in Indonesia. There are more reasons why our data might be an underestimation of the real incidence of ROP in Indonesia. Not all hospitals in Indonesia have an ophthalmologist, and therefore not all preterm infants are screened. Screening might not be according to the recommended schedule in all infants so that ROP can be missed even in screened infants. Infants might be too sick to be screened, and infants might not be screened after discharge.

Studies conducted in other LMIC up to 2015 also showed a higher incidence of ROP than in HIC. In addition, ROP was seen in infants with a higher gestational age and birth weight. A study from the Philippines showed a ROP incidence of 14% in all infants born before 36 weeks.[19] A small study from Brunei showed a prevalence of 35% in infants with a birth weight of 1300 ± 500 g and a gestational age of 29.5 ± 2.6 weeks.[20] In Thailand, a ROP incidence of 14% was found in infants with a mean birthweight of 1514 g and a gestational age of 31.8 weeks.[21] In India, ROP has been reported to occur in 21.7%– 51.9% of low birth weight infants. Most studies reported the mean birth weight of babies developing ROP to be above 1250 g and the incidence of severe ROP ranging from 5.0–44.9%.[22] In line with our findings for the period 2005-2015, these data indicate that ROP is prevalent in LMIC, including in infants with a higher birthweight and gestational age. A recent paper describes the current state of ROP in eight LMIC.[23] The incidence of ROP was not available for all countries. This incidence, mostly based on smaller studies in one institution, ranged from 14% to 50%. In almost all countries, infants up to 34 weeks and with a birthweight of 2000 g were screened. A study from Thailand, where only infants born <30 weeks and with a birthweight of <1500 g were screened, found a ROP incidence of 40%. In all countries, the screening rate was low, at <35%. The reasons

Confidential: For Review Only

mentioned for the high incidence of ROP was similar for all countries: a lack of awareness among paediatricians, a shortage of trained ophthalmologists and a lack of funds for screening. Almost all countries lacked oxygen delivery systems and oxygen saturation monitors. All countries fear an epidemic of blind infants as a result of ROP. In our view, the results of our survey indicate that it is possible to reduce the incidence of ROP, also in LMIC. The first step to stop this epidemic is to be aware of the risks of ROP. This concerns all those involved in the care of preterm infants, paediatricians, ophthalmologists, nurses and administrators.

Our study found that the screening rate for ROP is rather low in Indonesia, both in infants with a low gestational age and in infants with a higher gestational age who received supplemental oxygen for a prolonged period. This is most likely due to at least three factors: a lack of trained ophthalmologists, a lack of awareness among paediatricians of the importance of screening and a lack of funding for ophthalmologists. Paediatric ophthalmologists are mainly found in large academic hospitals and the national centres for perinatology. In almost all cases, there is only one paediatric ophthalmologist who is not always available. In order to increase the screening rate, paediatricians must be made aware of its importance and ophthalmologists must be trained to do it. Funds to carry out the screening need to be made available. It will not be possible to have, in a short period, enough trained ophthalmologists in Indonesia to have all preterm infants requiring ROP screening, screened according to the international accepted screening protocols. A potential solution to the lack of trained ophthalmologists might be cameras to make images of the retina and have these images evaluated by qualified, non-medical personnel. These assistants can send pictures of infants who might need ROP treatment via the internet to trained ophthalmologists. Simple, not expensive cameras have been developed. This system's

Confidential: For Review Only

advantage is that time required from ophthalmologists is reduced, and pictures can also be made in smaller hospitals without a trained ophthalmologist. This system is now implemented in India's parts, where it has been shown to be very effective. A sensitivity of 98% is achieved in detecting ROP cases that need intervention.[24,25]

We found no difference in the incidence of ROP in the institutions with a screening rate of at least 80%, compared to the whole group of infants. This indicates that a high number of infants are not screened while they develop ROP. The follow-up of preterm infants admitted to a NICU is low in Indonesia because of socioeconomic factors. It is not therefore known how often ROP was present in surviving, unscreened infants.

A lack of ophthalmologists trained in ROP screening is not a problem that is unique to Indonesia. There is a shortage of trained ophthalmologists in many LMIC and, where they do exist, screening is often not properly reimbursed and only the most committed ophthalmologists are willing to screen.[26]

In addition, paediatricians participating in this survey reported a reluctance among ophthalmologists to screen because of medico-legal problems, an imbalance between the level of difficulty and time spent examining very small premature babies and the results obtained, and the asynchronous examination fees in different health insurance systems. This all contrasts with the practice in HIC, where failure to screen an eligible infant for ROP could be considered malpractice.

The incidence of ROP in Harapan Kita Women and Children’s Hospital was significantly lower in the period 2016-2017 than in the period 2005-2015. At the same time, the screening rate improved to 93%. This indicates that ROP declined significantly at that hospital. In 2016, a strict policy was introduced to set the oxygen saturation monitor at 91% to 95% for preterm

Confidential: For Review Only

infants. The use of oxygen in the delivery room was also strictly regulated, with resuscitation of preterm infants starting at 30% oxygen. CPAP was given directly after birth and continued in the NICU. These measures are likely to have played an important role in reducing the incidence of ROP at that NICU.

The highest rate of both mortality and ROP was found in the university-based hospitals. These are referral hospitals that admit mothers with antenatal complications. They may therefore treat the sickest infants, sicker than those cared for in governmental and private hospitals. This could explain the higher rate of mortality and ROP. At the same time, however, the two national centres are also referral hospitals and they showed a lower mortality rate and a lower incidence of ROP (figures 1,2). This suggests that there could be other factors behind the higher incidence of ROP in the university-based hospitals. More liberal use of oxygen might be a cause. More studies are needed to determine the cause of the less favourable outcome in the university-based hospitals.

A limitation of this study is that the survey on the incidence of ROP as carried out in this study had not been done before. As a reference in this paper, we used data from one referral hospital as well as data from the literature. The rates of ROP found in one hospital in Indonesia[1] and the studies done in other centres[2] all showed a high incidence of ROP in the period before 2015. Therefore, we are convinced that the incidence of ROP was indeed much higher than suggested by the results of this survey. A second limitation of our survey is that we only included inborn infants. We do not have precise data on the ratio of inborn and outborn infants for all hospitals. In the Harapan Kita Hospital, one of the national referral hospitals, on average 71% of the admitted infants are inborn. We estimate that this percentage will be almost the same for the university-based NICUs. Unfortunately, there is

Confidential: For Review Only

no adequate neonatal transport service in Indonesia. Transportation is carried out by poorly trained personnel and only 100% oxygen can be given during transport. The referring and accepting neonatologists do not meet, making the transfer of information difficult and often incomplete.

Conclusion

The incidence of ROP in preterm infants in Indonesia was lower in 2016-2017 than in the period before 2015. This may be due to a higher awareness of ROP among practitioners and the introduction of a national healthcare plan. Our data, however, are likely to be an underestimation of the real incidence of ROP because of the higher mortality rate among small premature infants in Indonesia than in HIC and a low rate of screening and follow-up of surviving preterm infants.

Acknowledgements The authors would like to thank all infants and families whose data contributed to the IMSROP Study Group. We wish to thank our ophthalmologists Dr Habsyiyah, Dr Nani H. Widodo and Dr Florence Manurung. Together with all the ophthalmologists at each hospital included in this study, they were involved in examining patients and providing scientific input in the field of ophthalmology. We would also like to thank Prof. Rita S. Sitorus, who encouraged us to work together to reduce the incidence of ROP in Indonesia in terms of neonatology, ophthalmology and public health. We also thank all collaborators who are members of the IMSROP Study Group (Indonesian Multicenter Retinopathy of Prematurity) for providing data in this survey so that the entire data can be combined for analysis.

Confidential: For Review Only

Collaborators

*Members of the IMSROP (Indonesian Multicenter Retinopathy of Prematurity) Study Group: Rinawati Rohsiswatmo *(Rumah Sakit Cipto Mangunkusumo, Jakarta Pusat)*, Tetty Yuniati *(RSUP Hasan Sadikin, Bandung, Jawa Barat)*, Gatot Irawan Sarosa *(RSUP Dr Kariadi, Semarang, Jawa Tengah)*, Dwi Hidayah *(RSUD Dr Moewardi, Solo, Jawa Tengah),* Dina Angelika dan Risa Etika *(RSUD Dr Soetomo Surabaya, Jawa Timur)*, Eko Sulistijono *(RSUD Dr Syaiful Anwar Malang, Jawa Timur)*, Made Kardana *(RSUP Sanglah, Denpasar, Bali)*, Pertin Sianturi *(RSUP H. Adam Malik, Medan, Sumatera Utara)*, Eni Yantri *(RSUP Dr M. Djamil Padang, Sumatera Barat)*, Dewi Anggraini Wisnumurti *(RSUD Arifin Achmad Pekanbaru, Riau)*, Afifa Ramadanti *(RSUP M. Hoesin Palembang, Sumatera Selatan)*, Pudji Andayani *(RSUD Ulin Banjarmasin Kalimantan Selatan)*, Rocky Wilar *(RSUP Prof. Dr RD Kandou Hospital, Manado)*, Ema Alasiry (RSUP Dr Wahidin Sudirohusodo, Makasar, Sulawesi Selatan), Harris Alfan *(RS Atma Jaya, Jakarta Utara)*, Johanes Edy Siswanto *(RSAB Harapan kita dan RS Royal Taruma, Jakarta Barat)*, Naomi Esthernita *(Siloam Hospital Kebon Jeruk, Jakarta Barat)*, Fahrul Wakil Arbi *(RS Budi Kemuliaan, Jakarta Pusat)*, Nita Dewanti *(RS Premier Bintaro, Jakarta Selatan)*, Agnes Yuni Purwita Sari *(RSUP Persahabatan, Jakarta Timur)*, Ellen Sianipar *(RSUD Pasar Rebo Jakarta Timur)*, Robert Soetandio *(RS Awal Bros Hospital, Tangerang, Jawa Barat)*, Andika Tiurmaida *(RSUD Cibinong, Jawa Barat)*, Thomas Harry Adoe *(RSUD Kota Bekasi)*, Muda Isa Ariantana *(Santosa Hospital Bandung Cermai Bandung, Jawa Barat)*, Samad *(RSUP Dr Soeradji Tirtonegoro Klaten),* Anince *(RS Bakti Timah Karimun, Kepulauan Riau)*, Tri Putri Yuliani *(RSUD Siti Aisyah Kota Lubuk Linggau)*, Sumsel, Dina Frida *(RSUD Dr Sudarso, Kalimantan Barat dan RS Anugrah Bunda Khatulistiwa, Kalimantan Barat)*, Nurhandini Eka Dewi *(RSUD Mataram, Nusa Tenggara Barat)*, Woro Indri P. Purba *(RSUD Prof. Dr WZ Johannes Kupang, Nusa Tenggara Timur),* Sadiyah *(RSUD I Lagaligo, Luwu Timur, Sulawesi Selatan)*.

Contributors JES is the person directly involved in all aspects of this research and manuscript. JES, AB, PD and PJJS contributed to the study design and analysis. PJJS assisted in the literature review and wrote the report and the discussion section. AFB and PJJS are the guarantors for the study. JES, AB and PJJS had full access to the IMSROP data. All authors had full access to the summary data presented in this paper (including statistical reports and tables) and take responsibility for the integrity of the data and the accuracy of the data analysis.

Funding The authors have not declared a specific grant for this research from any funding agency in the public, commercial or not-for-profit sectors.

Confidential: For Review Only

Conflict of interests None declared.

Patient consent Detail has been removed from these case descriptions to ensure anonymity. The editors and reviewers have seen the detailed information available and are satisfied that the information backs up the case the authors are making.

Ethics approval The study was approved by the Institutional Review Board of the University of Indonesia School of Public Health (No. 32/H2.F10/PPM.00/13).

Provenance and peer review Not commissioned; externally peer-reviewed.

What is already known on this topic?

► Several studies in the previous decade in Indonesia showed a high incidence of ROP, which is a serious problem, as in other LMIC countries.

► The high rate of ROP in Indonesia is likely due to the expansion of neonatal intensive care, whereas there is a lack of awareness of the risks of developing ROP.

What does this study add?

► The incidence of ROP in Indonesia was lower in 2016-2017 than in surveys conducted before 2015.

► There was a difference in mortality and rate of ROP between the types of hospital. Both mortality and the incidence of ROP were highest in university-based hospitals.

► The lower rate of ROP could be the result of a new national guideline on ROP, an increased awareness following meetings and the introduction of national health care.

Confidential: For Review Only

References

1. Siswanto JE, Nani H Widodo NH, Sauer PJJ. *Arch Dis Child 2018*;103:619–21.
2. Siswanto JE, Sauer PJ. Retinopathy of prematurity in Indonesia: Incidence and riskfactors. J *Neonatal Perinatal Med* 2017;10:85–90.
3. Sitorus RS et al. POKJA Nasional ROP dan Bayi Prematur, Pedoman Nasional: Skrining dan Terapi ROP pada bayi prematur di Indonesia. eds 1. 2010
4. Gilbert C, Blencowe H. Retinopathy of prematurity: it is time to take action. *Comm Eye Health* 2017;99;45-48
5. Darlow BA, Gilbert CE, Quirogaet AM. Setting up and improving retinopathy of prematurity pros: interaction of neonatology, nursing and ophthalmology. *Clin Perinatol* 2013;40:215–27
6. Mataftsi A, Moutzouri S, Karagianni P, et al. Retinopathy of prematurity occurrence and evaluation of screening policy in a large tertiary Greek cohort. *Int Ophthalmol* 2020;40:385-91
7. Austeng D, Källen K, Hellström A, et al. Regional differences in screening for retinopathy of prematurity in infants born before 27 weeks of gestation in Sweden – the EXPRESS study. *Acta Ophthalmol* 2014; 92:311-5.
8. Draper ES, Zeitlin J, Field DJ, et al. Mortality patterns among very preterm babies: A comparative analysis of two European regions in France and England. *Arch Dis Child Fetal Neonatal Ed* 2007; 92: 356–360.
9. Milligan DW. Outcomes of children born very preterm in Europe. *Arch Dis Child Fetal Neonatal Ed* 2010;95:F234-40.
10. Draper ES, Zeitlin J, Fenton AC, et al, on behalf of the MOSAIC research group. Investigating the variations in survival rates for very preterm infants in 10 European regions: the MOSAIC birth cohort. *Arch Dis Child Fetal Neonatal Ed* 2009;3:F158-63.
11. Mohangoo AD, Buitendijk SE, Szamotulska K, et al. Gestational Age Patterns of Fetal and Neonatal Mortality in Europe: Results from the Euro-Peristat Project. *PLoS ONE* 2011;6:e24727.
12. Serenius F, Sjörs G, Blennow M, et al, and For the EXPRESS study group*. EXPRESS study shows significant regional differences in 1-year outcome of extremely preterm infants in Sweden. *Acta Paediatr* 2014;103:27–37.
13. Santhakumaran S, Statnikov Y, Gray D, et al, on behalf of the Medicines for Neonates Investigator Group. Survival of very preterm infants admitted to neonatal care in England 2008–2014: time trends and regional variation. *Arch Dis Child Fetal Neonatal Ed* 2018;103:F208–F215.
14. Norman M, Hallberg B, Abrahamsson T, et al. Association Between Year of Birth and 1-Year Survival Among Extremely Preterm Infants in Sweden During 2004-2007 and 2014-2016. *JAMA* 2019; 321:1188-1199.
15. Lee SK, McMillan DD, Ohlsson A, et al. Variations in practice and outcomes in the Canadian NICU network: 1996-1997. *Pediatrics* 2000;106:1070–9.

Confidential: For Review Only

16. Stoll BJ, Hansen NI, Bell EF, et al. Neonatal Outcomes of Extremely Preterm Infants From the NICHD Neonatal Research Network. *Pediatrics* 2010; 126: 443–456.
17. Stoll BJ, Hansen NI, Bell EF, et al. Trends in Care Practices, Morbidity, and Mortality of Extremely Preterm Neonates, 1993-2012. *JAMA* 2015;314:1039-51.
18. Su BH, Hsieh WS, Hsu CH, et al. Premature Baby Foundation of Taiwan (PBFT). Neonatal Outcomes of Extremely Preterm Infants From Taiwan: Comparison With Canada, Japan, and the USA. *Pediatr Neonatol* 2015;56:46-52.
19. Corpus K, Jimenez IV JM, Anzures R, Bascuna RI, Ventura R, Reandelar Jr M. Retinopathy of Prematurity Working Group. Proposed New Retinopathy of Prematurity Screening Criteria: Evidence for Including Older and Heavier Filipino Premature Babies. *Philipp J Ophthalmol* 2013;38:72-79.
20. Ali NAM, George J, Joshi N, et al. Prevalence of retinopathy of prematurity in Brunei Darussalam. *Int J Ophthalmol* 2013;3: 381–384.
21. Trinavarat A, Atchaneeyasakul L, Udompunturuk S. Applicability of American and British criteria for screening of retinopathy of prematurity in Thailand. *Jpn J Ophthalmol* 2004;48:50-3.
22. Dogra MR, Katoch D, Dogra M. An Update on Retinopathy of Prematurity (ROP). *Indian J Pediatr* 2017;12:930-6.
23. Bowe T, Nyamai L, Ademola-Popoola D, et al. The current state of retinopathy of prematurity in India, Kenya, Mexico, Nigeria, Philippines, Romania, Thailand, and Venezuela. *Digit J Ophthalmol* 2019;4:49–58.
24. Gilbert C, Wormald R, Fielder A, et al. Potential for a paradigm change in the detection of retinopathy of prematurity requiring treatment. *Arch Dis Child Fetal Neonatal Ed* 2016;101:F6-9.
25. Quinn GE, Vinekar A. The role of retinal photography and telemedicine in ROP screening. *Semin Perinatol* 2019;6:367-74.
26. Gilbert CE. Screening for retinopathy of prematurity: does one size fit all? *Arch Dis Child Fetal Neonatal Ed* 2016 Vol 101 No 4

Figure legend

Figure 1. The incidence of ROP based on gestational age in each group of hospitals in Indonesia 2016-2017.

Figure 2. Mortality based on the gestational age in each group of hospitals in Indonesia 2016-2017

Confidential: For Review Only

Table 1. Hospital-based ROP data surveillance in Indonesia 2016-2017 (GA ≤ 34 weeks)

Type of hospital	Total hospitals	Total infants	Survived	Died		Screened		ROP	
				n	%	n	%	n	%
RSCM/NRH	1	1038	890	148	14.3	392	44.0	7	1.8
HKWCHC	1	478	392	86	18.0	281	71.7	7	2.5
UBH	14	6549	4738	1811	27.7	2041	43.1	197	9.7
OH	18	4050	3261	789	19.5	711	21.8	17	2.4
- Government hospital	10	3020	2425	595	19.7	605	24.9	15	2.5
- Private hospital	8	1030	836	194	18.8	106	12.7	2	1.9
All hospitals	34	12115	9281	2834	23.4	3425	36.9	228	6.7

Note: RSCM/NRH: Ciptomangunkusumo Hospital (national referral hospital); HKWCH: Harapan Kita Women and Children's Hospital (National Centre for Women and Children's Health); UBH: university-based hospital; OH: other hospital; All: all hospitals; ROP; retinopathy of prematurity.

Confidential: For Review Only

Table 2a. ROP incidence in Harapan Kita Women and Children's Hospital, Indonesia based on the gestational age in 2005-2017

Variable		Infants GA <28 wks		Infants GA 28 - 32 wks		Infants GA >32 - 34 wks	
		2005-2015	2016-2017	2005-2015	2016-2017	2005-2015	2016-2017
Total (inborn infants)	n	185	54	569	221	NA	203
Died	n	91	27	126	24	-	24
	%	49	50	22	11	-	12
Survived	n	94	27	443	197	-	179
Screened	n	47	25	261	169	156	87
Screened/Survived	%	50	93	59	86	-	49
No ROP	n	28	23	187	167	126	84
ROP 1-2	n	9	2	66	1	29	3
ROP 3-5	n	10	0	8	1	1	0
Prevalence of Any ROP	%	40	8	28	1	19	3
Prevalence of Severe ROP	%	21	0	3	1	1	0

Note: ROP: Retinopathy of Prematurity; GA: gestational age; wks: weeks.

Confidential: For Review Only

Table 2b. ROP incidence in Indonesia based on the gestational age in 2016-2017

Variable		Infants GA <28 weeks					Infants GA 28 - 32 weeks					Infants GA >32-34 weeks				
		2016-2017					2016-2017					2016-2017				
		RSCM	HKWCH	UBH	OH	All	RSCM	HKWCH	UBH	OH	All	RSCM	HKWCH	UBH	OH	All
Total (inborn infants)	n	115	54	903	354	1426	501	221	2288	2044	5054	422	203	3358	1652	5635
Died	n	87	27	577	259	961	122	24	700	349	1195	26	24	534	181	765
	%	76	50	64	73	67	24	11	31	17	24	6	12	16	11	14
Survived	n	28	27	326	95	465	379	197	1588	1695	3859	396	179	2824	1471	4870
Screened	n	23	25	204	40	292	187	169	923	315	1594	182	87	914	356	1539
Screened/Survived	%	82	93	63	42	63	49	86	58	19	41	46	49	32	24	32
No ROP	n	22	23	161	33	239	185	167	751	309	1478	178	84	441	352	1480
ROP 1-2	n	1	2	34	5	42	2	1	94	6	103	2	3	45	2	52
ROP 3-5	n	0	0	9	2	11	0	1	12	0	13	2	0	3	2	7
Prevalence of Any ROP	%	4	8	21	18	18	1	1	11	2	7	2	3	5	1	3.8
Prevalence of Severe ROP	%	0	0	4	5	4	0	1	1	0	1	1	0	0	1	0.4

Note: RSCM/NRH: Ciptomangunkusumo Hospital (national referral hospital); HKWCH: Harapan Kita Women and Children Hospital (National Centre for Women and Children's Health); UBH: university-based hospital; OH: other hospital; All: all hospitals; ROP; Retinopathy of Prematurity; GA: gestational age.

Confidential: For Review Only

Table 3. Incidence of ROP in hospitals with 80% screening coverage in Indonesia, 2016-2017

Variable		Birth weight					Gestational age				
		<1000 g	1000-1500 g	>1500-2000 g	>2000-<2500 g	≥2500 g	<28 wks	28-32 wks	32-34 wks	34-<37 wks	≥37 wks
Total (inborn)	n	585	1541	2679	4522	20572	560	2144	2319	3913	20963
Died	n	394	449	303	163	266	391	508	244	165	267
	%	67	29	11	4	1	70	24	11	4	1
Survived	n	191	1092	2376	4359	20306	169	1636	2075	3748	20696
Screened	n	172	1040	1033	1186	14	168	1147	926	1199	5
Screened/Survived	%	90	95	43	27	0	99	70	45	32	0
No ROP	n	137	966	978	1159	14	132	1074	874	1169	5
ROP 1-2	n	28	57	53	27	0	27	62	46	30	0
ROP 3-5	n	7	17	2	0	0	9	11	6	0	0
Incidence of Any ROP	%	20	7	5	2	0	21	6	6	3	0
Incidence of Severe ROP	%	4	2	0	0	0	5	1	1	0	0

Note: these data come from 13 of 34 hospitals; ROP: retinopathy of prematurity

Confidential: For Review Only

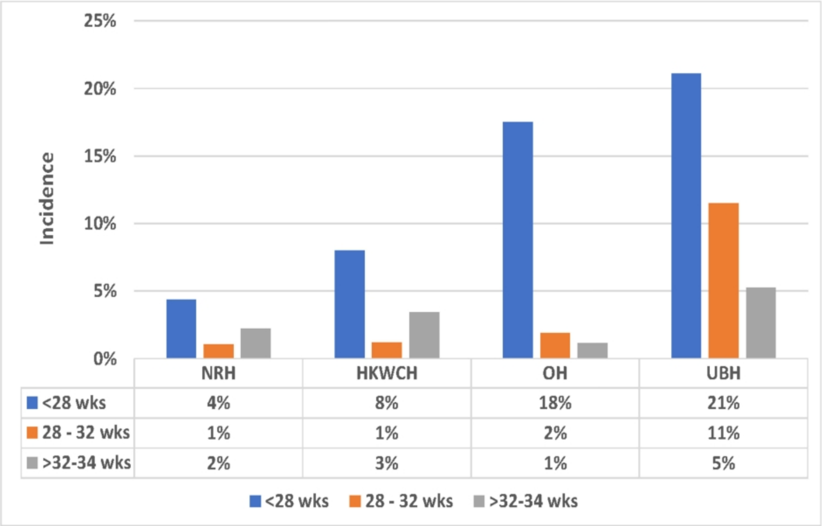

Note: NRH/RSCM: National Referral Hospital (Ciptomangunkusumo Hospital; HKWCH: Harapan Kita Women and Children Hospital (National Centre for Women and Children's Health); UBH: University Based Hospital; OH: Other Hospital; All: All Hospital; wks: weeks.

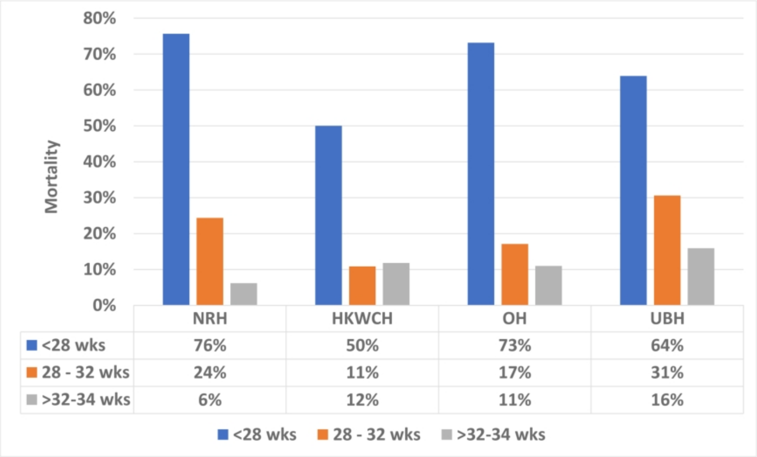

Note: RSCM/NRH: Ciptomangunkusumo Hospital (National Referral Hospital); HKWCH: Harapan Kita Women and Children Hospital (Health Centre); UBH: University Based Hospital; OH: Other Hospital.

148x105mm (1200 x 1200 DPI)

Supplementary Table. Incidence of ROP in Indonesia based on the birth weight in 2005-2017

Variable		Inborn infants BW <1000 gram						Inborn infants BW 1000-1500 gram						Inborn infants BW >1500-2000 gram					
		2005-2015	2016-2017					2005-2015	2016-2017					2005-2015	2016-2017				
		HKWCH	RSCM	HKWCH	UBH	OH	All Hospital	HKWCH	RSCM	HKWCH	UBH	OH	All Hospital	HKWCH	RSCM	HKWCH	UBH	OH	All Hospital
Total (Inborn)	n	182	132	71	1024	361	1588	437	306	160	2104	1094	3664	748	475	236	3730	2055	6496
Died	n	85	101	38	567	256	962	72	93	24	640	346	1103	.	42	24	532	205	803
	%	47	77	54	55	71	61	16	30	15	30	32	30	.	9	10	14	10	12
Survived	n	97	31	33	457	105	626	365	213	136	1464	748	2561	.	433	212	3198	1850	5693
Screened	n	59	26	29	197	35	287	252	169	123	802	273	1413	163	177	54	1131	495	1857
Screened/Survived	%	61	84	88	43	33	46	69	79	90	55	36	55	.	41	25	35	27	33
No ROP	n	27	24	26	156	28	234	172	167	120	696	265	1248	139	175	53	488	492	1208
ROP 1-2	n	22	2	2	33	5	42	71	1	3	89	7	100	22	1	1	54	2	58
ROP 3-5	n	10	0	1	6	2	9	9	1	0	17	1	19	2	1	0	0	1	2
Prevalence of Any ROP	%	54	8	10	20	20	18	32	1	2	13	3	8	15	1	2	5	1	3
Prevalence of Severe ROP	%	17	0	3	3	6	3	4	1	0	2	0	1	1	1	0	0	0	0

Note: RSCM/NRH: Ciptomangunkusumo Hospital (National Referral Hospital); HKWCH: Harapan Kita Women and Children Hospital (National Centre for Women and Children's Health); UBH: University Based Hospital; OH: Other Hospital; All: All Hospital; ROP; Retinopathy of Prematurity; BW: Birth weight.

Confidential: For Review Only
